# Microfluidic high-throughput optimization enables scalable synthesis of high-entropy fluorophosphate cathode

**DOI:** 10.1093/nsr/nwag195

**Published:** 2026-03-27

**Authors:** Zhicheng Tian, Yuanzheng Zhou, Xude Yu, Yadi Zhao, Simeng Liu, Yuxin Chen, Xingjiang Wu, Zhuo Chen, Jianhong Xu

**Affiliations:** State Key Laboratory of Chemical Engineering and Low-Carbon Technology, Department of Chemical Engineering, Tsinghua University, Beijing 100084, China; State Key Laboratory of Chemical Engineering and Low-Carbon Technology, Department of Chemical Engineering, Tsinghua University, Beijing 100084, China; State Key Laboratory of Chemical Engineering and Low-Carbon Technology, Department of Chemical Engineering, Tsinghua University, Beijing 100084, China; State Key Laboratory of Chemical Engineering and Low-Carbon Technology, Department of Chemical Engineering, Tsinghua University, Beijing 100084, China; State Key Laboratory of Chemical Engineering and Low-Carbon Technology, Department of Chemical Engineering, Tsinghua University, Beijing 100084, China; State Key Laboratory of Chemical Engineering and Low-Carbon Technology, Department of Chemical Engineering, Tsinghua University, Beijing 100084, China; National-Local Joint Engineering Laboratory for Energy Conservation in Chemical Process Integration and Resources Utilization, School of Chemical Engineering and Technology, Hebei University of Technology, Tianjin 300130, China; State Key Laboratory of Chemical Engineering and Low-Carbon Technology, Department of Chemical Engineering, Tsinghua University, Beijing 100084, China; State Key Laboratory of Chemical Engineering and Low-Carbon Technology, Department of Chemical Engineering, Tsinghua University, Beijing 100084, China

**Keywords:** microfluidic high-throughput optimization, large-scale synthesis, high-entropy fluorophosphate cathode, sodium-ion batteries, high rate capacity

## Abstract

Large-scale synthesis of high-entropy fluorophosphate cathode for high-rate sodium-ion batteries remains a huge challenge due to time-consuming conditional optimization and poor phase purity. Herein, for the first time, we overcome the bottlenecks of large-scale synthesis of high-entropy Na_3_V_1.9_M_0.1_(PO_4_)_2_F_3_ (HE-NVPF) cathode libraries by a microfluidic high-throughput optimization (MHO) strategy. Due to precise regulation and *in situ* monitoring of nucleation-growth kinetics, the microfluidic *in situ* Raman spectrometer achieves high iteration efficiency of conditional optimization with 400 times increase over traditional strategies. Consequently, kilogram-scale HE-NVPF with high phase purity is rapidly synthesized within 2 h, which delivers stable multielectron transfer, superior Na^+^ diffusion kinetics and negligible volume expansion/contraction, exhibiting reversible phase transition and excellent structural ruggedness. As a result, the representative Na_3_V_1.9_(Ca, Mg, Zr, Mn, Cr)_0.1_(PO_4_)_2_F_3_ cathode presents record-breaking rate capacity (108.6 mAh g^−1^ at 50 C), large energy density (371.9 Wh kg^−1^) and good cycling stability. This MHO strategy can be extended to synthesize other high-entropy Na_3_V_1.9_M_0.1_(PO_4_)_2_F_3_, such as M = (Mg, Zr, Co, Mn, Cr), (Zr, Ca, Fe, Mn, Cr), (Mg, Ca, Ni, Mn, Cr), (Zr, Cu, Mg, Mn, Cr) and (Ga, Zr, Ca, Mn, Cr). Our work provides a novel insight into machine intelligent synthesis of high-entropy materials and facilitates their industrialized process.

## Teaser

This work develops a microfluidic high-throughput optimization strategy, which enables rapid, kilogram-scale synthesis of high-performance sodium-ion battery cathodes, offering a novel insight into machine intelligent production of energy materials.

## INTRODUCTION

Establishing grid-scale energy storage systems is pivotal for marketable development of electric vehicles, portable electronics, low-altitude economy and artificial intelligence (AI) [1–4]. Among energy-storage devices, sodium-ion batteries (SIBs) have emerged as promising candidates for grid-scale energy storage systems, driven by their cost-effectiveness, inherent safety and resource sustainability [5–7]. As a burgeoning cathode material of SIBs, the high-entropy sodium vanadium fluorophosphates [Na_3_V_1.9_M_0.1_(PO_4_)_2_F_3_; HE-NVPFs] have attracted enormous attention because of their good electron conductivity, abundant ion diffusion pathways and remarkable redox activity [8–12]. However, the large-scale synthesis of HE-NVPFs for high-rate SIBs remains a significant challenge. According to the reaction mechanism, the synthesis of HE-NVPFs involves a nucleation-growth process to form polyoxovanadate intermediates and subsequent high-temperature calcination [8]. When considering large-scale synthesis, due to multicomponent diversity and sluggish atomic diffusion, the polyoxovanadate intermediates tend to be thermodynamically unstable, thus usually suffering from severe phase segregation and resulting in low phase purity of HE-NVPFs during high-temperature calcination [13]. In fact, the low phase purity will impede charge transfer and Na^+^ diffusion kinetics, which leads to irreversible phase transition and poor structural stability during the charge/discharge process, significantly reducing the rate capacity [14,15]. Therefore, the large-scale synthesis of high-phase-purity HE-NVPFs with high-rate capacity urgently requires systematic conditional optimization of the nucleation-growth process.

Traditional strategies, such as the solid-state reaction and sol–gel method, are frequently used to synthesize gram-scale HE-NVPFs together with infrequent optimization of reaction conditions [8,10,11,16–18]. In this respect, the solid-state reaction involves high-speed ball milling of gel-state polyoxovanadate intermediates and subsequent high-temperature calcination of HE-NVPFs [17]. Due to enhanced bulk conductivity and robust structural stability, the gram-scale Na_3+x_V_1.76−x_Zn_x_(GaCrAlIn)_0.06_(PO_4_)_3_ presents a good rate capacity of 90.17 mAh g^−1^ at 60 C [17]. Moreover, the sol–gel method is broadly reported to synthesize gel-state polyoxovanadate intermediates by uninterrupted stirring, evaporating and further calcination into HE-NVPFs [8]. For example, the gram-scale Na_3_V_1.9_(Ca, Mg, Al, Cr, Mn)_0.1_(PO_4_)_2_F_3_ [8] and Na_3_V_1.94_(Cr, Mn, Co, Ni, Cu)_0.06_(PO_4_)_3_O_2_F [10] exhibit excellent rate capacities of 71.4 and 60.9 mAh g^−1^ at 50 C, respectively, because of the elevated mean voltage plateau and enhanced Na-storage kinetics. However, several challenges remain in traditional strategies: (i) for the electrochemical performance, the gram-scale HE-NVPFs with unoptimized phase purity prepared by those traditional strategies usually possess a rate capacity of less than 100 mAh g^−1^ when the rate increases to above 50 C, thus limiting their high-power density applications; (ii) for the synthesis process, due to poor controllability, those traditional strategies fail to regulate and monitor in real time the reactant evolution and nucleation-growth kinetics in solution [19]. Generally, the traditional conditional optimization is mainly realized by individually changing reaction time and reaction temperature, accompanied by low iteration efficiency (one reaction condition more than 1 h) and a time-consuming process. As a result, when considering large-scale synthesis of HE-NVPFs, in those traditional strategies it is difficult to screen optimal reaction conditions to suppress phase segregation of thermodynamically unstable polyoxovanadate intermediates, usually leading to low phase purity during high-temperature calcination [13]. Consequently, developing a high-throughput optimization strategy with high iteration efficiency to identify optimal conditions and obtain stable polyoxovanadate clusters, thereby inhibiting phase segregation and obtaining high-phase-purity HE-NVPFs, is of great significance for high-rate SIBs.

Herein, for the first time, we propose a microfluidic high-throughput optimization (MHO) strategy to break the bottlenecks of large-scale synthesis of HE-NVPF cathode libraries. The home-made microfluidic *in situ* Raman spectrometer is developed to enable precise regulation and *in situ* monitoring of nucleation-growth kinetics, achieving a higher iteration efficiency (>400 reaction conditions within 1 h) than that of traditional strategies (1 reaction condition more than 1 h) [20–25]. Based on high-throughput optimization, the polyoxovanadate clusters with dynamic stability can be accurately prepared, which can effectively suppress phase segregation and therefore endow HE-NVPFs with high phase purity. The aberration-corrected scanning transmission electron microscopy STEM (AC-STEM) and X-ray absorption spectroscopy (XAS) reveal high phase purity and specific coordination environments of HE-NVPFs. Remarkably, the high phase purity enables stable multielectron transfer, superior Na^+^ diffusion kinetics and negligible volume expansion/contraction, exhibiting reversible phase transition and excellent structural ruggedness, which is confirmed by density functional theory (DFT) calculations and *in situ* X-ray diffraction (XRD) analysis. As a representative, the kilogram-scale Na_3_V_1.9_(Ca, Mg, Zr, Mn, Cr)_0.1_(PO_4_)_2_F_3_ cathode is rapidly produced within 2 h, which presents a large specific capacity of 121.8 mAh g^−1^ at 0.5 C, a record-breaking rate capacity of 108.6 mAh g^−1^ at 50 C, excellent cycling stability (86.0% capacity retention after 500 cycles at 1 C) and a high energy density of 371.9 Wh kg^−1^. More notably, this MHO strategy can be extended to synthesize other HE-NVPFs, such as M = (Mg, Zr, Co, Mn, Cr), (Zr, Ca, Fe, Mn, Cr), (Mg, Ca, Ni, Mn, Cr), (Zr, Cu, Mg, Mn, Cr) and (Ga, Zr, Ca, Mn, Cr), indicating its high universality. This MHO strategy opens up new opportunities for conditional optimization and machine intelligent synthesis of kilogram-scale high-entropy materials.

## RESULTS

**Figure 1.**
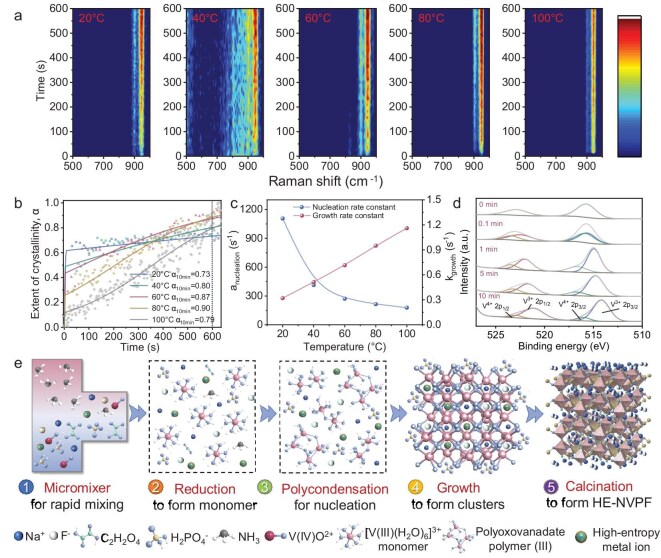
The high-throughput conditional optimization of an HE-NVPF. (a) The time-resolved *in situ* Raman spectra of the HE-NVPF under various reaction temperatures. (b) Time-resolved crystallinity extent (α) curves of the HE-NVPF under various reaction temperatures. (c) Nucleation-growth rate constants of the HE-NVPF under various reaction temperatures. (d) *Ex situ* XPS spectra of the HE-NVPF under various residence times at 80°C. (e) Schematic illustration of nucleation-growth mechanism of the HE-NVPF.

Currently, the systematic optimization of reaction conditions with high iteration efficiency can provide theoretical guidance for large-scale synthesis of HE-NVPFs [26,27]. Traditional solid-state reaction and sol–gel methods usually suffer from poor controllability, failing to regulate or monitor in real time the reactant evolution in solution and nucleation-growth kinetics, and therefore produce low iteration efficiency for optimization of reaction conditions (one reaction condition more than 1 h) (Fig. S1). To realize high-throughput conditional optimization with high iteration efficiency, we design a home-made microfluidic *in situ* Raman spectrometer that can eliminate the effects of mass/heat transfer on nucleation-growth reaction and monitor in real time the reactant evolution in solution (Figs S2 and S3). Figure 1a displays the time-resolved *in situ* Raman spectra of nucleation-growth process under various reaction temperatures at 20, 40, 60, 80 and 100°C. To minimize calculation errors, the strongest characteristic peak at 930–970 cm^−1^ is utilized to characterize the specific reactant evolution process, which quickly appears in *in situ* Raman spectra, corresponding to the V–O–V stretching vibration of polyoxovanadate [27,28]. With the increase of reaction time, the characteristic peak intensities gradually reach equilibrium, indicating the formation of polyoxovanadate clusters with good dynamic stability [29–32].

Moreover, the specific iteration efficiency is also investigated by time-resolved *in situ* Raman spectra. Figure 1b presents the time-resolved crystallinity extent (α) curves of polyoxovanadate clusters under various reaction temperatures, which are quantitatively calculated by normalized integration of characteristic peaks via Equation S1 [26,27]. Notably, the home-made microfluidic *in situ* Raman spectrometer can obtain more than 400 reaction conditions within 1 h under various reaction times (0–10 min) and reaction temperatures (20, 40, 60, 80 and 100°C), which exhibits a 400× increase of iteration efficiency over traditional strategies (1 reaction condition more than 1 h). Based on the crystallinity extent of polyoxovanadate under 400 reaction conditions, the reaction time and temperature are simultaneously optimized. Obviously, as the reaction time increases, the crystallinity extent first increases abruptly within 10 s (an ultrafast nucleation process to form polyoxovanadate) and then gradually increases until reaching equilibrium at 10 min (a gradual growth process to form polyoxovanadate clusters), indicating the optimal reaction time of 10 min. Moreover, the optimal reaction temperature is also analyzed by comparing crystallinity extents at 10 min. Notably, with the increase of reaction temperature from 20°C to 80°C, the crystallinity extent increases from 74% to 90%. When the reaction temperature increases from 80°C to 100°C, the crystallinity extent decreases from 90% to 80%, indicating the optimal reaction temperature of 80°C. Undoubtedly, by high-throughput conditional optimization with high iteration efficiency, we obtain the optimized reaction temperature (80°C) and residence time (10 min) within 1 h, which can achieve the highest crystallinity extent (α > 90%) of polyoxovanadate clusters (Fig. 1b).

Based on the Gualtieri model in Equation S2 [33], the nucleation-growth rate constants under various reaction temperatures are calculated. As displayed in Fig. 1c, when the reaction temperature increases from 20°C to 100°C, the nucleation rate constant (a_nucleation_) decreases from 1107 to 178 s^−1^, and the growth rate constant (k_growth_) increases from 0.32 to 1.16 s^−1^. This result confirms the promoting effect of the reaction temperature on the growth process and the inhibitory effect of the reaction temperature on the nucleation process [34,35]. Moreover, the corresponding nucleation-growth activation energies are further calculated by the Arrhenius equation (Equation S3) via nucleation-growth rate constants [27]. As depicted in Fig. S4, the fitting result exhibits low activation energies of 14.18 kJ mol^−1^ in the nucleation process and 13.97 kJ mol^−1^ in the growth process, demonstrating the intrinsic nucleation-growth kinetics. Furthermore, the *ex situ* X-ray photoelectron spectroscopy (XPS) spectra are also employed to explore the effect of reaction time on the nucleation-growth reaction at 80°C (Fig. 1d) [36]. Obviously, the high-resolution V 2p XPS spectra present a rapid nucleation-growth reaction of polyoxovanadate clusters, which is highly consistent with *in situ* Raman spectra.

Enabled by high-throughput conditional optimization with high iteration efficiency, the specific nucleation-growth mechanism of HE-NVPFs is further revealed (Fig. 1e). Specifically, the reaction solutions are injected into a T-shaped micromixer, which induces a rapid reduction reaction from the V(IV)O^2+^ precursor to the [V(III) (H_2_O)_6_]^3+^ monomer (Equation S4) [29]. Notably, early on the C_2_H_2_O_4_ reduces NH_4_VO_3_ into the V(IV)O^2+^ precursor during preparation of the salt solution. In the microfluidic microchannel, the ammonia water further initiates fast hydrolysis and polycondensation nucleation of the [V(III) (H_2_O)_6_]^3+^ monomer to form polyoxovanadate as the crystal nucleus [37,38]. Subsequently, the crystal nucleus with low interfacial energy can provide growth sites for heterogeneous crystallization of PO_4_^3−^, F^−^, Na^+^ and high-entropy metal ions to produce polyoxovanadate clusters [37,38]. Remarkably, the polyoxovanadate clusters with good dynamic stability can effectively suppress phase reorganization and separation of HE-NVPFs [13]. Meanwhile, the high-entropy metal ions will occupy the same Wyckoff position in the HE-NVPF via charge balance to produce a single-phase structure [39]. Obviously, the high-throughput conditional optimization with high iteration efficiency can provide theoretical guidance for subsequent kilogram-scale synthesis of the HE-NVPF.

**Figure 2.**
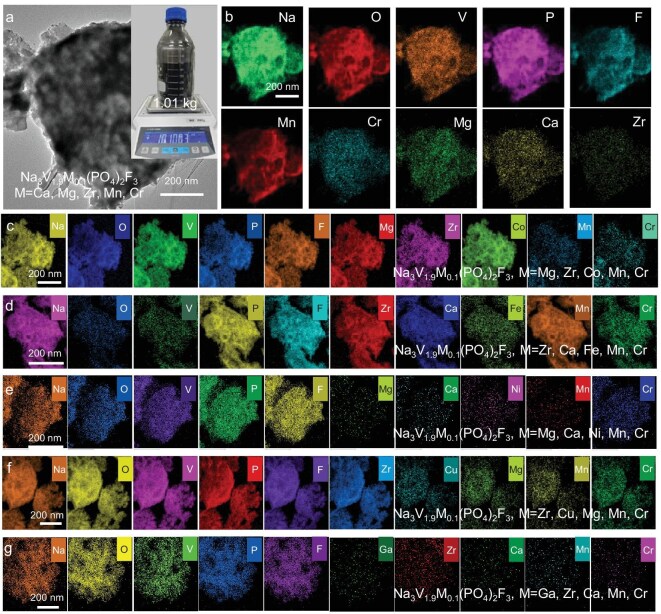
Kilogram-scale synthesis of an HE-NVPF and its morphology characterizations. (a) Kilogram-scale Na_3_V_1.9_(Ca, Mg, Zr, Mn, Cr)_0.1_(PO_4_)_2_F_3_ and its TEM image. (b-g) EDS mappings of Na_3_V_1.9_(Ca, Mg, Zr, Mn, Cr)_0.1_(PO_4_)_2_F_3_ (b), Na_3_V_1.9_(Mg, Zr, Co, Mn, Cr)_0.1_(PO_4_)_2_F_3_ (c), Na_3_V_1.9_(Zr, Ca, Fe, Mn, Cr)_0.1_(PO_4_)_2_F_3_ (d), Na_3_V_1.9_(Mg, Ca, Ni, Mn, Cr)_0.1_(PO_4_)_2_F_3_ (e), Na_3_V_1.9_(Zr, Cu, Mg, Mn, Cr)_0.1_(PO_4_)_2_F_3_ (f), and Na_3_V_1.9_(Ga, Zr, Ca, Mn, Cr)_0.1_(PO_4_)_2_F_3_ (g).

Based on the theoretical guidance of the high-throughput conditional optimization with high iteration efficiency, the kilogram-scale HE-NVPF libraries are synthesized via the MHO strategy. As shown in Fig. S5, the MHO strategy consists of a T-shaped micromixer for ultra-rapid mixing and reduction reactions, a microfluidic microchannel for rapid nucleation growth reactions, spray drying for fast evaporation drying and a tube furnace for high-temperature calcination. As a representative, Na_3_V_1.9_(Ca, Mg, Zr, Mn, Cr)_0.1_(PO_4_)_2_F_3_ is first selected to investigate the effect of residence time on high-entropy architecture. As shown in Fig. S6, when the residence time is 1 min, the nucleation-growth reaction does not reached equilibrium, and therefore the polyoxovanadate with thermodynamic instability will inevitably suffer from thermodynamically driven phase reorganization and separation during spray drying and the calcination process. Accordingly, the irregular morphology, uneven size distribution, severe phase segregation and poor element uniformity emerge. When the nucleation-growth reaction reaches equilibrium at 10 min, the polyoxovanadate clusters with good dynamic stability and well-distributed elements can suppress thermodynamically driven phase segregation during spray drying and calcination process [16,40]. Therefore, the polyoxovanadate clusters exhibit regular morphology, homogeneous size distribution and good element uniformity (Fig. S7). Benefiting from the rapid nucleation-growth reaction and fast evaporation, the formation of polyoxovanadate clusters with good dynamic stability (rate-determining step for synthesizing HE-NVPFs) is greatly intensified by the MHO strategy. Therefore, the Na_3_V_1.9_(Ca, Mg, Zr, Mn, Cr)_0.1_(PO_4_)_2_F_3_ with a high yield of 1.01 kg is achieved (Fig. 2a). More notably, the elements of Na, O, V, P, F, Ca, Mg, Zr, Mn and Cr are uniformly distributed in Na_3_V_1.9_(Ca, Mg, Zr, Mn, Cr)_0.1_(PO_4_)_2_F_3_ (10 min), indicating the successful synthesis of an HE-NVPF (Fig. 2b). By analyzing the effect of residence time on high-entropy architecture, we confirm that the polyoxovanadate clusters with good dynamic stability serve as an important precondition for large-scale synthesis of HE-NVPFs.

To further confirm the universality in large-scale synthesis of HE-NVPF libraries, the NVPFs with various high-entropy metal-ion combinations are also produced, such as M = (Mg, Zr, Co, Mn, Cr), (Zr, Ca, Fe, Mn, Cr), (Mg, Ca, Ni, Mn, Cr), (Zr, Cu, Mg, Mn, Cr) and (Ga, Zr, Ca, Mn, Cr). As shown in Fig. S8, all XRD diffraction peaks can be indexed to tetragonal crystals of NASICON-type NVPF characteristics with P42/mnm structure (PDF#89-8485) [20], which validates the universal synthesis of various HE-NVPFs. In addition, all high-resolution transmission electron microscopy (HRTEM) images show well-defined lattice fringes with no other impurities, confirming the single-phase nature with high phase purity of various HE-NVPFs. More notably, all energy-dispersive X-ray spectroscopy (EDS) mapping images of various HE-NVPFs deliver uniform element distribution, such as (Na, O, V, P, F, Mg, Zr, Co, Mn, Cr), (Na, O, V, P, F, Zr, Ca, Fe, Mn, Cr), (Na, O, V, P, F, Mg, Ca, Ni, Mn, Cr), (Na, O, V, P, F, Zr, Cu, Mg, Mn, Cr) and (Na, O, V, P, F, Ga, Zr, Ca, Mn, Cr), which further verify their high-entropy characteristics (Fig. 2c–g, Table S1). Consistent with these findings, inductively coupled plasma atomic emission spectroscopy (ICP-OES) analysis (Tables S2–S7) further confirms the uniform elemental compositions of HE-NVPF libraries. Those remarkable advantages affirm that the kilogram-scale HE-NVPF libraries possess high phase purity and uniform element distribution, which may provide novel guidance for large-scale synthesis of high-entropy polyanionic cathode materials.

**Figure 3.**
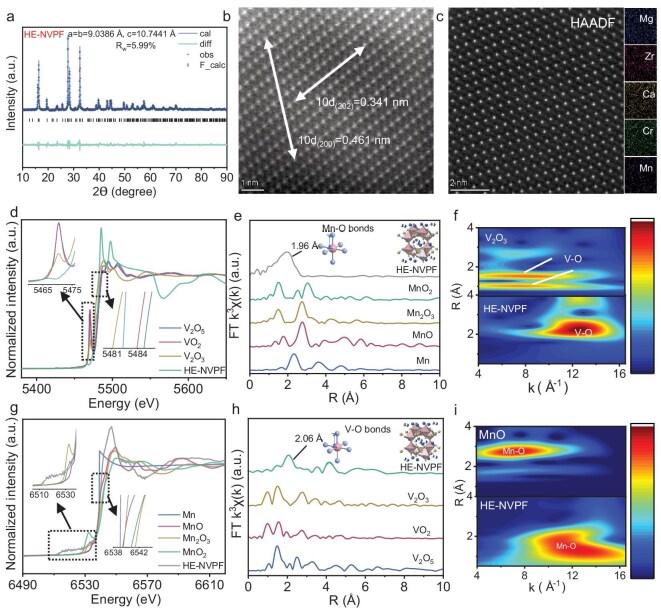
Chemical and structural characterizations of an HE-NVPF. (a) Rietveld-refinement XRD spectrum of an HE-NVPF. (b and c) AC-STEM images and corresponding EDS mapping of the HE-NVPF. (d) Normalized XANES spectrum at the V K-edge of the HE-NVPF. (e) V K-edge FT-EXAFS k^3^χ(k) spectrum of the HE-NVPF. (f) V K-edge WT contour plots for EXAFS k^3^χ(k) of the HE-NVPF. (g) Normalized XANES spectrum at the Mn K-edge of the HE-NVPF. (h) Mn K-edge FT-EXAFS k^3^χ(k) spectrum of the HE-NVPF. (i) Mn K-edge WT contour plots for EXAFS k^3^χ(k) of the HE-NVPF.

As a typical representative of HE-NVPF, Na_3_V_1.9_(Ca, Mg, Zr, Mn, Cr)_0.1_(PO_4_)_2_F_3_ is utilized to conduct chemical and structural characterizations. In this section, the pristine NVPF and medium-entropy Na_3_V_1.9_(Zr, Mn, Cr)_0.1_(PO_4_)_2_F_3_ (ME-NVPF) are also prepared using the MHO strategy to serve as chemical and structural comparisons. Rietveld refinement of XRD spectra is first conducted to analyze crystal structure and phase purity of NVPF, ME-NVPF and HE-NVPF. As illustrated in Fig. 3a and Figs S9 and S10, all XRD peaks can be well indexed to the tetragonal crystal of NASICON-type NVPF characteristics with a P42/mnm structure (PDF#89-8485) [20], indicating the successful synthesis of high-crystallinity NVPF, ME-NVPF and HE-NVPF. Compared to NVPF (876.32 Å^3^) and ME-NVPF (876.41 Å^3^), HE-NVPF has a larger unit cell volume of 877.75 Å^3^, demonstrating that high-entropy substitution with various metal-ion sizes can produce lattice distortion, which facilitates multi-electron transfer and Na^+^ diffusion kinetics [8] (Table S8). The HRTEM images are also employed to analyze crystal structure and phase purity. As shown in Fig. S11, the HE-NVPF presents clear diffraction points and well-defined lattice fringes with interlayer spacing of 0.32 nm, confirming the high crystallinity and phase purity [41]. Moreover, the clear diffraction points, well-defined lattice fringes and uniform element distribution also appear in the NVPF and ME-NVPF, indicating the success of universal synthesis (Figs S12–S15). For the first time, the crystal structure and phase purity of HE-NVPFs are investigated by AC-STEM images in Fig. 3b and c. Notably, the well-defined lattice fringes with interlayer spacing of 0.34 and 0.46 nm point to the (202) and (200) planes of the HE-NVPF, respectively. Furthermore, the highly ordered atomic configuration and homogeneous element distribution further verify the high-entropy nature, high crystallinity and phase purity of the HE-NVPF. Additionally, the specific elemental ratio of Mg:Ca:Zr:Cr:Mn in the HE-NVPF is 1.0:1.1:1.0:1.0:1.0, suggesting that the equimolar doping of each element can provide high configurational entropy for excellent electrochemical energy-storage behavior [13] (Table S2).

The XPS spectra are employed to explore element components and the electronic configuration. As displayed in Figs S16–S18, compared with the NVPF and ME-NVPF, the survey XPS spectrum of the HE-NVPF reveals additional high-entropy elements of Ca, Mg, Zr, Mn and Cr, proving the successful high-entropy substitution into the central V atom in the NVPF crystal structure. Figures S19–S21 show high-resolution V 2p XPS spectra of the NVPF, ME-NVPF and HE-NVPF. Notably, the HE-NVPF possesses two characteristic peaks at 515.5 and 522.9 eV belonging to the V^3+^ 2p_3/2_ and V^3+^ 2p_1/2_ orbits, respectively, which is highly coincident with XRD analysis [42,43]. Moreover, the characteristic peaks of the HE-NVPF at lower binding energies compared to the NVPF and ME-NVPF confirm that high-entropy substitution can regulate local electronic configuration and therefore result in multi-electron transfer and decreased electron density [44]. The high-resolution Mn 2p XPS spectrum of the HE-NVPF is also analyzed in Fig. S22. Obviously, the HE-NVPF has a characteristic peak of the Mn^2+^ 2p_1/2_ orbit at 641.4 eV, affirming the presence of the Mn element after high-entropy substitution [45].

XAS is used to analyze the coordination environments and chemical bonds of the HE-NVPF. As shown in Fig. 3d, the X-ray absorption near edge structure (XANES) spectrum at the V K-edge of the HE-NVPF shows an absorption threshold close to that of V_2_O_3_, indicating the V^3+^ in the HE-NVPF. Compared with V_2_O_3_ (5470.3 eV), HE-NVPF pre-edge peak moves to a lower energy at 5466.3 eV, suggesting that high-entropy substitution changes V^3+^ coordination environments and facilitates 1s → 3d orbital transition [46,47]. Moreover, the variations in V^3+^ coordination environments are also studied by Fourier transforms of the extended X-ray absorption fine structure (FT-EXAFS), shown in Fig. 3e. Interestingly, the HE-NVPF presents a larger V–O bond distance of 2.06 Å in the R space than that of V_2_O_3_ (1.50 Å) because of high-entropy substitution and the inductive effect induced via V–O–P bonds [36,48–50]. The increased V–O bond distance suggests a more open and flexible polyanionic framework, which reduces the electrostatic repulsive forces during Na^+^ migration [8,11,36]. Analogously, the wavelet transform (WT) contour plots of the HE-NVPF show higher k-values in the V–O bond than in V_2_O_3_, further confirming the variations in coordination environments (Fig. 3f). As a proof of high-entropy substitution, the XANES spectrum at the Mn K-edge of the HE-NVPF is further discussed. Notably, the Mn K-edge of the HE-NVPF exhibits an absorption threshold close to that of MnO, indicating that Mn exists predominantly in the +2 oxidation state. This conclusion is consistent with the high-resolution Mn 2p XPS spectrum [51]. Similar to the V K-edge, the HE-NVPF pre-edge peak in the Mn K-edge XANES spectrum also presents a lower energy at 6519.8 eV than that of MnO (6534.9 eV) (Fig. 3g). Also, the HE-NVPF shows a shorter Mn–O bond distance of 1.96 Å in the FT-EXAFS spectrum and higher k-values in WT contour plots than in MnO (Mn–O bond distance of 2.76 Å) (Fig. 3h and i). These results certify that high-entropy substitution with various metal-ion sizes will cause variations in coordination environments due to lattice distortion and electron orbital transition [13]. In general, the MHO strategy endows HE-NVPFs with high crystallinity and phase purity, which can provide high Na^+^ diffusion kinetics in subsequent electrochemical characterizations.

**Figure 4.**
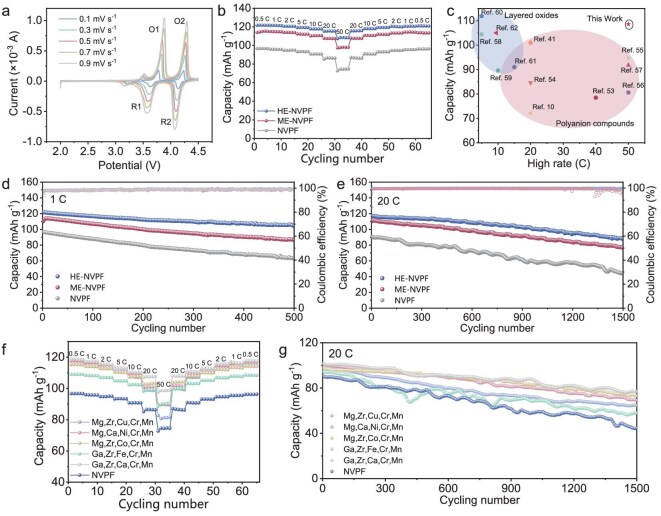
Electrochemical characterizations of an HE-NVPF in a half-cell. (a) CV curves of the HE-NVPF under various scanning rates. (b) Rate capacities of NVPF, ME-NVPF and HE-NVPF under various current rates. (c) Rate capacity comparison of the HE-NVPF by the MHO strategy with reported SIB cathodes by conventional methods. (d) Cycling performance of NVPF, ME-NVPF and HE-NVPF at 1 C. (e) Cycling performance of NVPF, ME-NVPF and HE-NVPF at 20 C. (f) Rate capacities of HE-NVPF libraries under various current rates. (g) Cycling performance of HE-NVPF libraries at 20 C.

Considering the chemical and structural superiorities, the Na^+^ diffusion kinetics of NVPFs, ME-NVPFs and HE-NVPFs are also comprehensively evaluated. The Na^+^ diffusion kinetics are firstly analyzed by cyclic voltammetry (CV) curves. As shown in Fig. 4a and Figs S23 and S24, the HE-NVPF exhibits two oxidation peaks at 3.78 (O1) and 4.23 V (O2) corresponding to Na^+^ dissociation reactions, and two reduction peaks at 3.64 (R1) and 4.14 V (R2) corresponding to Na^+^ insertion reactions. Among these, the O1/R1 peaks correspond to relatively slow Na^+^ dissociation reactions, while the high-potential process controlled by the surface active site is ascribed to fast electron transfer [52]. Notably, the HE-NVPF exhibits lower oxidation–reduction peak potential difference of 0.11 V than the NVPF (0.14 V) and ME-NVPF (0.16 V), indicating that high-entropy substitution can decrease electrode polarization due to the enhanced multi-electron transfer and Na^+^ diffusion kinetics [53]. Based on the Randles–Sevcik equation (Equation S5), the linear relationships between scan rates and peak currents are calculated, as shown in Fig. S25. Obviously, the higher b-values and linearity of the HE-NVPF compared to that of the NVPF and ME-NVPF imply higher pseudocapacitive contribution during the Na^+^ dissociation/insertion reaction process [41]. The specific pseudocapacitive contributions under various scan rates are also calculated. As illustrated in Fig. S26, the HE-NVPF possesses a higher pseudocapacitive contribution than the NVPF, attributed to enhanced Na^+^ storage reaction kinetics.

Moreover, the galvanostatic charge–discharge (GCD) curves of the HE-NVPF show well-defined voltage plateaus at 3.78 and 4.23 V during Na^+^ dissociation reactions and 3.64 and 4.14 V during Na^+^ insertion reactions, which agree well with the CV results (Fig. S27). Notably, the HE-NVPF presents a high reversible capacity of 121.7 mA h g^−1^ at 0.5 C, higher than the NVPF (96.7 mA h g^−1^) and ME-NVPF (114.4 mA h g^−1^) (Figs S28 and S29). After 60 cycles at various rates, the HE-NVPF maintains the same capacity comparable to its initial value, indicating the rapid and reversible Na^+^ de/insertion process and low polarization phenomenon (Fig. 4b) [8]. Especially, the record-breaking rate capacity of 108.6 mA h g^−1^ at 50 C of the HE-NVPF by the MHO strategy is much higher than that of reported SIB cathodes by conventional methods (Fig. 4c) [11,41,54–63].

Generally, the long-term cycling stability is a vital evolution indicator of high-performance SIB cathodes. Therefore, the cycle performances of the NVPF, ME-NVPF and HE-NVPF are systematically compared. As shown in Fig. 4d, the HE-NVPF presents higher capacity retention of 86% at 1 C after 500 cycles than the NVPF (65.4%) and ME-NVPF (75.4%), indicating the good structural ruggedness and electrochemical reversibility. Even at high current rates of 20 C, the HE-NVPF still maintains a high capacity retention of 75.2% after 1500 cycles (Fig. 4e). However, the NVPF and ME-NVPF only retain low capacity retention of 49.1% and 70.0%, respectively. In addition, the average capacity loss per cycle of the HE-NVPF is 0.028% at 1 C and 0.016% at 20 C, which are obviously lower than those of NVPF (0.069% at 1 C and 0.034% at 20 C) and ME-NVPF (0.049% at 1 C and 0.020% at 20 C).

To confirm the universality in kilogram-scale synthesis of HE-NVPF libraries, the Na^+^ storage performance of NVPF with various high-entropy metal-ion combinations is also characterized. Notably, the various HE-NVPF combinations exhibit various rate and cycle performances, due to the diversity in electronic structure and redox activity that affect ion diffusion kinetics and structural stability, which are caused by various ionic radii and electrochemical activities of various doping elements [8,10,64–67]. As depicted in Fig. 4f, the Na_3_V_1.9_(Mg, Zr, Cu, Cr, Mn)_0.1_(PO_4_)_2_F_3_, Na_3_V_1.9_(Mg, Ca, Ni, Cr, Mn)_0.1_(PO_4_)_2_F_3_, Na_3_V_1.9_(Mg, Zr, Co, Cr, Mn)_0.1_(PO_4_)_2_F_3_, Na_3_V_1.9_(Ga, Zr, Fe, Cr, Mn)_0.1_(PO_4_)_2_F_3_ and Na_3_V_1.9_(Ga, Zr, Ca, Cr, Mn)_0.1_(PO_4_)_2_F_3_ present larger reversible capacities of 82.72, 89.57, 89.45, 90.22, 83.0 and 86.74 mA h g^−1^ at 50 C than the NVPF (74.5 mA h g^−1^), indicating that the various HE-NVPFs with high phase purity possess superior Na storage performance because of rapid multi-electron transfer and Na^+^ diffusion kinetics. More importantly, after 1500 cycles at 20 C, the Na_3_V_1.9_(Mg, Zr, Co, Mn, Cr)_0.1_(PO_4_)_2_F_3_, Na_3_V_1.9_(Zr, Ca, Fe, Mn, Cr)_0.1_(PO_4_)_2_F_3_, Na_3_V_1.9_(Mg, Ca, Ni, Mn, Cr)_0.1_(PO_4_)_2_F_3_, Na_3_V_1.9_(Zr, Cu, Mg, Mn, Cr)_0.1_(PO_4_)_2_F_3_ and Na_3_V_1.9_(Ga, Zr, Ca, Mn, Cr)_0.1_(PO_4_)_2_F_3_ still maintain higher capacity retention of 65.6%, 69.0%, 75.1%, 61.5% and 74.8% than the NVPF (49.1%), demonstrating the superior structural ruggedness (Fig. 4g). Those remarkable advantages affirm that the kilogram-scale synthesized HE-NVPF cathode libraries with high phase purity possess excellent Na^+^ diffusion performance.

**Figure 5.**
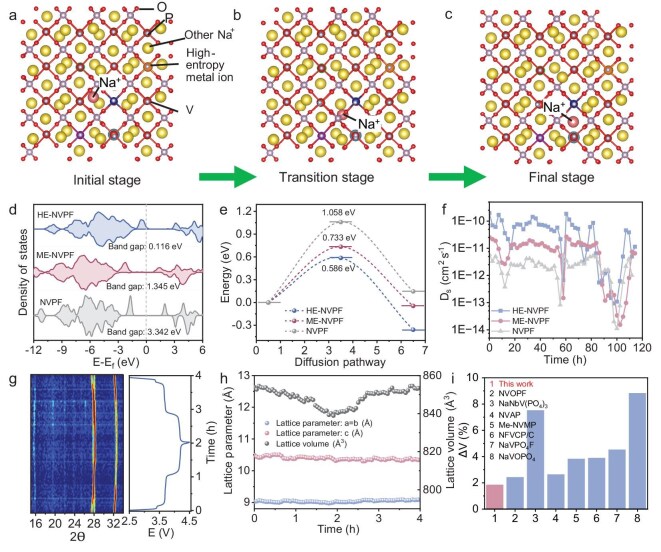
DFT calculations and *in situ* XRD analysis on Na^+^ migration kinetics of an HE-NVPF. (a–c) The schematic illustrations of Na^+^ migration pathways in the HE-NVPF under various stages. (d) DOS of NVPF, ME-NVPF and HE-NVPF. (e) The Na⁺ migration energy barriers of NVPF, ME-NVPF and HE-NVPF. (f) Na⁺ diffusion coefficients of NVPF, ME-NVPF and HE-NVPF. (g) 2D contour plot of electrochemical *in situ* XRD and corresponding GCD curves of HE-NVPF during the first cycle at 0.5 C. (h) Specific lattice parameters of the HE-NVPF via electrochemical *in situ* XRD. (i) The lattice parameter comparison of the HE-NVPF by the MHO strategy with reported polyanionic cathodes by conventional methods.

To thoroughly reveal the excellent Na^+^-storage performance of high-phase-purity HE-NVPFs, DFT calculations are conducted to investigate Na^+^ migration energy barriers and energy bandgap. As shown in Fig. 5a–c and Figs S30 and S31, all geometry configurations of NVPFs, ME-NVPFs and HE-NVPFs show close total energies, indicating stable configurations in realistic situations. In general, the NVPF, ME-NVPF and HE-NVPF lattices contain immobile Na (1) sites and active Na (2) sites for Na^+^ dissociation/insertion reactions. Among those, the Na (1) sites refer to two sites that are fully occupied by Na^+^, and the Na (2) sites refer to two sites that are partly occupied by Na ions [8]. During the charge–discharge process, Na^+^ will gradually migrate from active Na (2) sites to immobile Na (1) sites, which involves an initial stage, a transition stage and a final stage. Moreover, the electron localization function (ELF) is performed to reveal the local electronic environment around Na sites (Fig. S32). Compared to the NVPF and ME-NVPF, the HE-NVPF possesses more intense electron localization around Na sites, suggesting that high-entropy substitution can regulate the local electronic environment and therefore accelerate interfacial multi-electron transfer around Na sites. Density of states (DOS) calculations are also conducted to confirm the variations in local electronic environment. As displayed in Fig. 5d, the NVPF shows discontinuous bandgaps with a high energy bandgap of 3.342 eV around the Fermi level, indicating poor electrical conductivity. When doping Zr, Mn and Cr elements, the ME-NVPF exhibits weak bandgap continuity with a decreased energy bandgap of 1.345 eV around the Fermi level, implying improved electrical conductivity because of the partially excited electrons from the valence band to the conduction band. After high-entropy substitution, the abundant interfacial multi-electrons are excited from the valence band to the conduction band in the HE-NVPF, and therefore exhibit dazzling bandgap separation with the lowest energy bandgap of 0.116 eV around the Fermi level, demonstrating excellent electrical conductivity. In fact, the high electrical conductivity enhanced by high-entropy substitution can greatly decrease the Na^+^ migration energy barriers. In the calculated model, the dopant positions are randomly distributed, and the chosen path is representative of the active high-entropy regions where the substitution effect is most pronounced, which can be used to accurately calculate the Na^+^ migration energy barriers. As expected, in Fig. 5e, the HE-NVPF presents the lowest Na^+^ migration energy barriers of 0.586 eV, compared to the NVPF (1.058 eV) and ME-NVPF (0.733 eV), further confirming that high-entropy substitution with multi-electron transfer can effectively increase the Na^+^ diffusion kinetics.

The specific Na^+^ diffusion coefficients (D_s_) of NVPFs, ME-NVPFs and HE-NVPFs are also calculated by galvanostatic intermittent titration technique (GITT) measurements (Figs S33–S35). As shown in Fig. 5f, the HE-NVPF displays the largest Na^+^ diffusion coefficients compared to the NVPF and ME-NVPF, manifesting the optimal Na^+^ diffusion kinetics via high-entropy substitution, which is highly consistent with DFT calculation results. To reveal the structure–activity relationship between crystal structure evolution and the Na^+^ dissociation/insertion reaction during the charge–discharge process, electrochemical *in situ* XRD of the HE-NVPF is conducted for the first cycle at 0.5 C. As shown in Fig. 5g, the (024) and (116) diffraction peaks slightly move to higher degree during the 2 h charge process, indicating the Na^+^ dissociation reaction in the HE-NVPF crystal structure. In the subsequent 2 h discharge process, the (024) and (116) diffraction peaks reversibly return to the initial low degree, corresponding to Na^+^ insertion reactions in the HE-NVPF crystal structure. Significantly, the HE-NVPF crystal structure exhibits no obvious phase transition during Na^+^ dissociation/insertion reactions, demonstrating that high-entropy substitution can endow the HE-NVPF crystal structure with excellent structural ruggedness and electrochemical reversibility, similar to previous XRD results [8]. Based on electrochemical *in situ* XRD curves, the lattice parameters are calculated to further verify excellent structural ruggedness and electrochemical reversibility. As calculated in Fig. 5h, the lattice volume of the HE-NVPF first decreases in the charge process and then increases to the initial value in the discharge process, ascribed to reversible Na^+^ dissociation/insertion reactions. Notably, the HE-NVPF by the MHO strategy undergoes a negligible volume change (ΔV) of 1.8%, which is much lower than that of reported polyanionic cathodes by conventional methods (Fig. 5i), such as NVOPF (2.4%) [41], NaNbV(PO_4_)_3_ (7.5%) [68], NVAP (2.62%) [11], Me-NVMPs (3.8%) [12], NFVCP/C (3.87%) [69], NaVPO_4_F (4.5%) [70] and NaVOPO_4_ (8.8%) [71]. Undoubtedly, the HE-NVPF synthesized by the MHO strategy with high phase purity exhibits excellent Na^+^ storage performance because of low Na^+^ migration energy barriers and high electrical conductivity.

**Figure 6.**
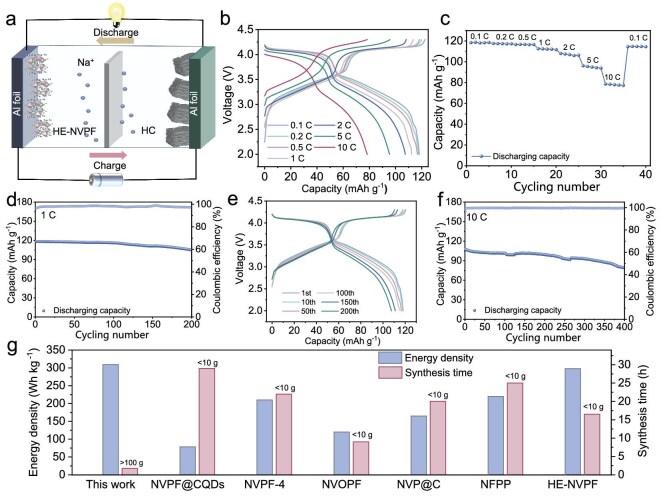
Electrochemical characterizations of an HE-NVPF in an HE-NVPF//HC full cell. (a) Schematic illustration of the HE-NVPF//HC full cell. (b) GCD curves of the HE-NVPF//HC full cell under various current rates. (c) Rate capacities of the HE-NVPF//HC full cell under various current rates. (d) Cycling performance of the HE-NVPF//HC full cell at 1 C. (e) GCD curves of the HE-NVPF//HC full cell under various cycles. (f) Cycling performance of the HE-NVPF//HC full cell at 10 C. (g) Comparison of energy density and synthesis time.

The kilogram-scale HE-NVPF synthesized by the MHO strategy with high phase purity displays great potential in full-cell applications, which is also an important parameter to elucidate the practicability of cathode. As shown in Fig. 6a, the HE-NVPF//hard carbon (HC) full cell is constructed by Na_3_V_1.9_(Ca, Mg, Zr, Mn, Cr)_0.1_(PO_4_)_2_F_3_ as cathode and commercial HC as anode. The GCD curves are employed to evaluate Na storage performance of the HE-NVPF//HC full cell. Obviously, the GCD curves of the HE-NVPF//HC full cell present a shape similar to an HE-NVPF half cell under various current rates, implying the well-matched HE-NVPF and HC (Fig. 6b). As shown in Fig. 6c, the HE-NVPF//HC full cell displays high reversible capacities of 118.3, 117.6, 116.9, 112.1, 108.0, 96.2 and 78.6 mA h g^−1^ at 0.1, 0.2, 0.5, 1, 2, 5 and 10 C, respectively. Notably, the high-rate capacity of 118.3 mA h g^−1^ at 0.1 C is mainly attributed to rapid Na^+^ diffusion kinetics and reversible Na^+^ dissociation/insertion reactions. Moreover, the reversible Na^+^ dissociation/insertion reactions are confirmed by long-term cycling stability in Fig. 6d. After 200 cycles at 1 C, the HE-NVPF//HC full cell still retains 88.9% initial capacity. Meanwhile, the corresponding GCD curves of the HE-NVPF//HC full cell present almost no obvious change after 1, 10, 50, 100, 150 and 200 cycles, suggesting a low polarization phenomenon and excellent structural ruggedness of the HE-NVPF (Fig. 6e). Figure 6f exhibits long-term cycling stability of the HE-NVPF//HC full cell at 10 C under 400 cycles. Remarkably, the high capacity retention of 73.7% is still obtained, further revealing the good cycling stability. Furthermore, the energy density of the HE-NVPF//HC full cell and synthesis time of the HE-NVPF are synthetically evaluated. As calculated in Fig. 6g, the HE-NVPF//HC full cell by the MHO strategy possesses a higher energy density of 371.9 Wh kg^−1^ than those of reported polyanionic full cells by conventional methods, such as NVPF@CQDs (170 Wh kg^−1^) [72], NVPF-4 (350 Wh kg^−1^) [73], NVOPF (220 Wh kg^−1^) [74], NVP@C@3DPGFs-NS (320 Wh kg^−1^) [75], NFPP (242 Wh kg^−1^) [76] and HE-NVPFs (351 Wh kg^−1^) [8]. Due to high yield (>0.2 kg h^−1^) via the MHO strategy, the HE-NVPF presents the lowest synthesis time among those of conventional methods, such as NVPF@CQDs (29 h), NVPF-4 (22 h), NVOPF (9 h), NVP@C (20 h), NFPP (25 h) and HE-NVPFs (16.5 h). In conclusion, the kilogram-scale HE-NVPF synthesized by the MHO strategy with high phase purity demonstrates promising potential for application in commercial SIB full cells.

## CONCLUSION

In summary, for the first time, we break the bottlenecks of large-scale synthesis of HE-NVPF cathode libraries via an MHO strategy, such as M = (Ca, Mg, Zr, Mn, Cr), (Mg, Zr, Co, Mn, Cr), (Zr, Ca, Fe, Mn, Cr), (Mg, Ca, Ni, Mn, Cr), (Zr, Cu, Mg, Mn, Cr) and (Ga, Zr, Ca, Mn, Cr). The microfluidic *in situ* Raman spectrometer exhibits a 400× increase of iteration efficiency on conditional optimization over traditional strategies, which enables kilogram-scale synthesis of HE-NVPFs with high phase purity within 2 h. Benefitting from stable multielectron transfer, superior Na^+^ diffusion kinetics and negligible volume expansion/contraction, the high-phase-purity HE-NVPF delivers reversible phase transition and excellent structural ruggedness, which is demonstrated by AC-STEM, XAS, DFT and *in situ* XRD characterization. As a result, the representative Na_3_V_1.9_(Ca, Mg, Zr, Mn, Cr)_0.1_(PO_4_)_2_F_3_ cathode presents a record-breaking rate capacity, excellent cycling stability and high energy density. This is the first report for machine intelligent synthesis of large-scale HE-NVPF libraries, and therefore facilitates their potential industrial production.

## METHODS

### Synthesis procedures

The microfluidic high-throughput strategy includes a T-shaped micromixer, a microfluidic microchannel, spray drying and a calcination process. For representative synthesis of Na_3_V_1.9_(Ca, Mg, Zr, Mn, Cr)_0.1_(PO_4_)_2_F_3_, the NH_4_VO_3_ (Mreda), C_2_H_2_O_4_ (Aladdin), NH_4_H_2_PO_4_ (Mreda), NaF (Aladdin), Ca(NO_3_)_2_.4H_2_O (3AChem), Mg(NO_3_)_2_.6H_2_O (Mreda), Zr(NO_3_)_3_·5H_2_O (3AChem), Cr(NO_3_)_3_·9H_2_O (Aladdin), MnC_4_H_6_O_4_·4H_2_O (Mreda) in calculated stoichiometric ratio were dissolved in distilled water to obtain the salt solution. The ammonia water and salt solution were then injected into the T-shaped micromixer for rapid mixing and reduction reactions (pH = 7). Subsequently, the mixed flow was continuously injected into the microfluidic microchannel to synthesize homogeneous stable polyoxovanadate clusters via a nucleation-growth process under 80°C water-bath conditions for 10 min. Afterwards, the reaction flow containing homogeneous stable polyoxovanadate clusters was continuously processed into submicron-scale microspheres by a spray dryer. Optimized parameters included inlet/outlet temperatures of 140°C/90°C, a feed rate of 10 mL min^−1^, an atomization pressure of 0.5 MPa, and a gas flow rate of 1200 L h^−1^. Lastly, the Na_3_V_1.9_(Ca, Mg, Zr, Mn, Cr)_0.1_(PO_4_)_2_F_3_ was obtained by high-temperature calcination at 550°C for 1.5 h, and the synthesis yield was calculated for 0.2 kg h^−1^.

The other HE-NVPFs were synthesized by the same experimental procedure except for changing high-entropy metal-ion combinations. The NVPF was synthesized by the same experimental procedure without adding high-entropy metal-ion sources. The ME-NVPF was synthesized by the same experimental procedure except for adding three metal-ion sources of Zr(NO_3_)_3_·5H_2_O (3AChem), Cr(NO_3_)_3_·9H_2_O (Aladdin) and MnC_4_H_6_O_4_·4H_2_O (Mreda).

### Electrochemical characterization

The electrochemical characterizations were performed in a CR2032 coin cell. The electrode slurry was prepared by mixing the HE-NVPF, acetylene black and polyvinylidene fluoride (PVDF) (7:2:1 weight ratios) in *N*-methylpyrrolidone (NMP, Aladdin), which was coated onto a 12 mm-diameter aluminum foil, with an active material mass loading of 1.5–2 mg cm^−2^. Subsequently, the half-cells were assembled by using an HE-NVPF, ME-NVPF and NVPF as cathode, sodium foil (Aladdin) as anode, glass fiber filter paper (Whatman) as separator and 1.0 M NaPF6-EC/DMC/EMC (DoDoChem) as electrolyte in an Ar-filled glovebox. The full cells were assembled by using HC as anode, with the other part the same as the half-cells and the N/P ratio of the full cell was set as 1.7–1.8. The HC anode slurry was prepared by mixing HC, acetylene black and PVDF in a weight ratio of 7:2:1 using NMP as the solvent. The slurry was coated onto aluminum foil, and electrodes were punched into disks with a diameter of 14 mm, with a mass loading of 0.9–1.2 mg cm^−2^. The HC electrodes then underwent an electrochemical pre-sodiation process in a half-cell configuration, involving five charge/discharge cycles at a current density of 0.1 A g^−1^ before the full cell was assembled. All electrochemical tests were carried out using a LAND CT3200A cycler. GITT measurements were then conducted after the three precycles at 0.1 C with a titration step at 0.1 C of 15 min and a relaxation step of 2 h. CV testing was performed on a CHI760E electrochemical workstation. Electrochemical impedance spectroscopy (EIS) was also performed on a CHI760E electrochemical workstation.

## Supplementary Material

nwag195_Supplemental_File
