# Supplementary material for: Microfluidic high-throughput optimization enables scalable synthesis of high-entropy fluorophosphate cathode
Source: Natl Sci Rev. 2026 Mar 27;13(10):nwag195. doi: 10.1093/nsr/nwag195 (PMC13192515; doi:10.1093/nsr/nwag195)
Supplement: nwag195_Supplemental_File [file nwag195_supplemental_file.pdf]

Supplementary data for

Microfluidic high-throughput optimization enables scalable synthesis of high-entropy fluorophosphate cathode

Zhicheng Tian[1,+], Yuanzheng Zhou[1,+], Xude Yu[1], Yadi Zhao[1], Simeng Liu[1], Yuxin Chen[1], Xingjiang Wu[2,*], Zhuo Chen[1] and Jianhong Xu[1,*]

[1]State Key Laboratory of Chemical Engineering and Low-Carbon Technology, Department of Chemical Engineering, Tsinghua University, Beijing 100084, China;

[2]National-Local Joint Engineering Laboratory for Energy Conservation in Chemical Process Integration and Resources Utilization, School of Chemical Engineering and Technology, Hebei University of Technology, Tianjin 300130, China

***Corresponding authors.** E-mails: xujianhong@tsinghua.edu.cn; wuxingjiang@hebut.edu.cn

[+]Equally contributed to this work.

Supplemental Methods

Material characterization

In situ Raman spectroscopy was performed using a LabRAM HR Evolution confocal Raman spectrometer. The solid samples were characterized using X-ray diffraction (XRD) on a Rigaku SmartLab X-ray diffractometer. Sample morphologies were examined through scanning electron microscopy (Zeiss Sigma 300). Transmission electron microscopy (TEM) with energy-dispersive X-ray spectroscopy (EDS), were conducted using a transmission electron microscope(JEOL JEM-2100) operating to analyze particle size and morphology. HAADF imaging and atomic-resolution EDS mapping were performed using an FEI Titan 80-300 field-emission aberration-corrected STEM. X-ray photoelectron spectroscopy (XPS) measurements were performed on a VG MultiLab 2000 system. ICP-OES analysis was performed using a Thermo Scientific™ QC ICP-OES. Electrochemical in-situ XRD characterization was performed using a beryllium (Be) electrode on a Panalytical X'Pert3 Powder diffractometer, with testing conducted at 0.5 C after activation via three cycles at 0.1 C. The XAFS measurements were performed at the Taiwan Photon Source (TPS), China, a third-generation synchrotron light source operated at 1.5 GeV.

Design of home-made microfluidic in-situ Raman spectrometer

The home-made microfluidic in-situ Raman spectrometer contains T-shaped micromixer, microfluidic reaction chamber and in-situ Raman spectrometer. The inner diameter of commercial T-shaped micromixer was 450 μm. The microfluidic reaction chamber with 10 mm length, 10 mm width and 1mm depth was manufactured by automatic Relief Carving Machine（YB4030P）. Then, the T-shaped micromixer and microfluidic reaction chamber were connected by microchannel with inner diameter of 1 mm. The salt solution and ammonia water were injected into T-shaped micromixer by microfluidic pump. At last, the in-situ Raman spectra of polyoxovanadate clusters were obtained by using a LabRAM HR Evolution confocal Raman spectrometer with a 532 nm laser source and a 10× objective lens.

Computational details

The electronic structure of high-entropy doping $Na_3V_2(PO_4)_2F_3$ was explored by spin-polarized density functional theory (DFT) calculations via the Quickstep module of CP2K 7.1 program package[1,2]. Due to the low concentration of high-entropy dopants, a large supercell of $Na_3V_2(PO_4)_2F_3$ containing 288 atoms was firstly built. Then, some V atoms were randomly substituted by the doping atoms (Mg, Ca, Cr, Mn, Zr) to present the effect of high-entropy doping[3]. Next, geometry optimization was performed under PBEsol level[4,5]. In detail, the SCF iteration was solved via the orbital transformation (OT) method, and the structure was fully relaxed until the maximum forces exerted on each atom < 0.0001 Hartree/Bohr[6]. The convergence threshold of the SCF iteration was 1×10^{-7} Hartree, the energy cutoff was set as 1200 Ry and the Brillouin zone was sampled with 1x1x1 k-point mesh. Besides, the climbing image nudged elastic band (CI-NEB) method was used to obtain the migration path of Na[7]. Finally, to handle the self-interaction error due to the strongly correlated d electrons and obtain accurate electronic structures, hybrid DFT calculations were conducted using HSE06 functional[8,9]. The basis set was as follows: TZV2P-MOLOPT-GTH for (O, F, P) and TZVP-MOLOPT-SR-GTH for (Na, Mg, Ca, V, Cr, Mn, Zr). The auxiliary density matrix method (ADMM) was employed to accelerate hybrid DFT calculations and the details were as follows: pFIT3 basis for (O, F, P, Na, Mg) and FIT11 basis for (Ca, V, Cr, Mn, Zr)[10]. The analyses of density of states (DOS), electron localization function (ELF) and electron density were done with the help of Multiwfn 3.8 (dev) and VESTA 3.90.5a[11,12].

Supplemental Figures

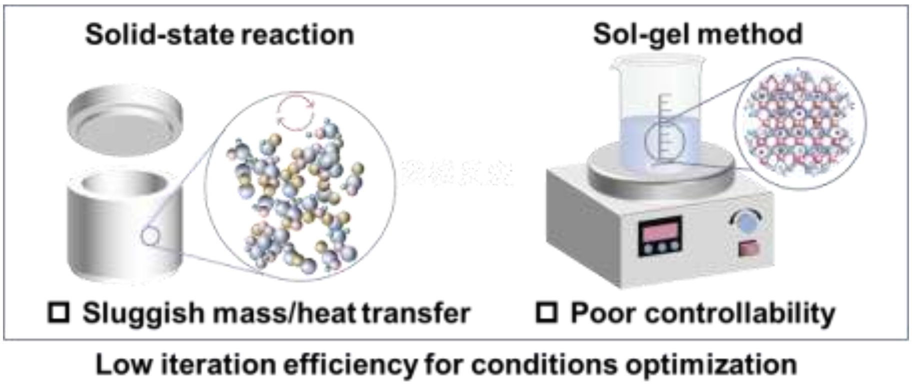

Fig. S1. Schematic of solid-state reaction and sol-gel method.

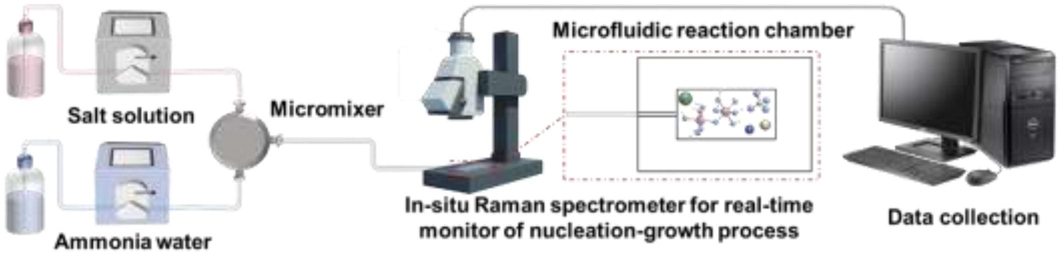

Fig. S2. Schematic illustration of the home-made microfluidic in-situ Raman spectrometer.

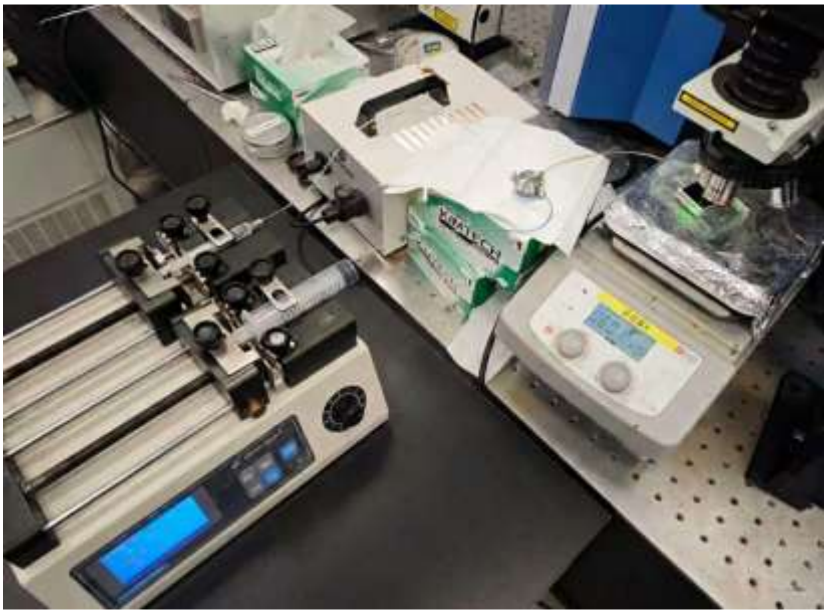

Fig. S3. Digital photographs of the actual home-made microfluidic in-situ Raman spectrometer.

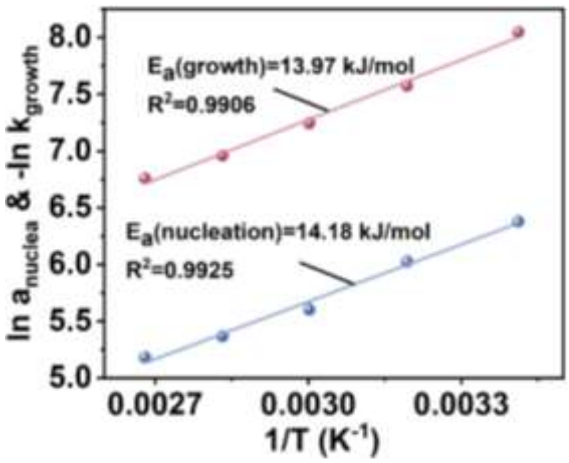

Fig. S4. The nucleation-growth activation energies of polyoxovanadate clusters.

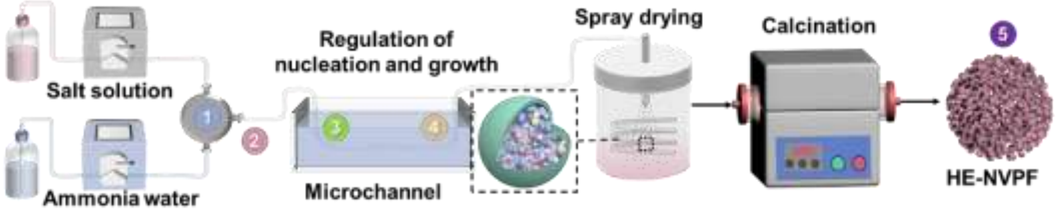

Fig. S5. Schematic illustration of universal scalable synthesis of HE-NVPF via MHO strategy.

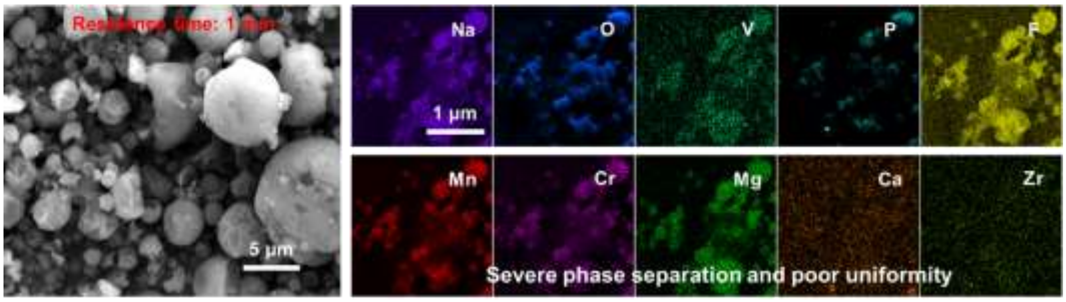

Fig. S6. The SEM image and EDS mappings of polyoxovanadate prepared at the residence time of 1 min.

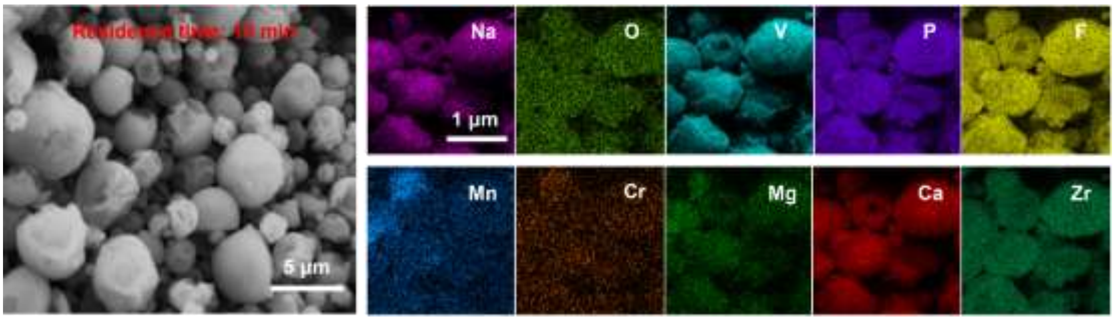

Fig. S7. The SEM image and EDS mappings of polyoxovanadate clusters prepared at the residence time of 10 min.

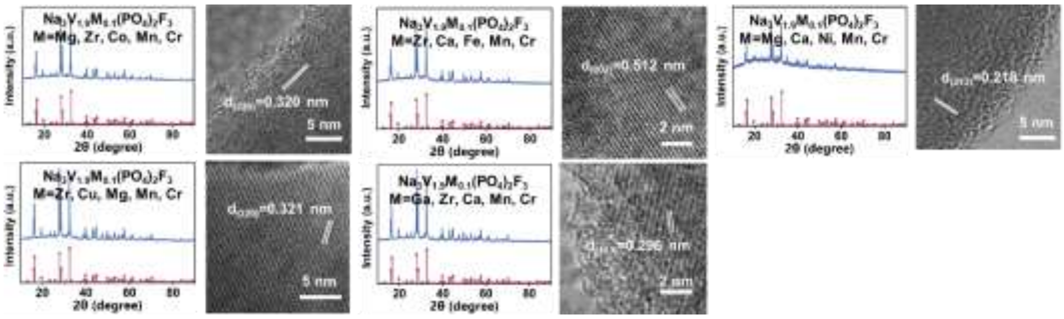

Fig. S8. The Rietveld refinement XRD spectra and HRTEM images of high-entropy $Na_3V_{1.9}M_{0.1}(PO_4)_2F_3$ (HE-NVPF) libraries.

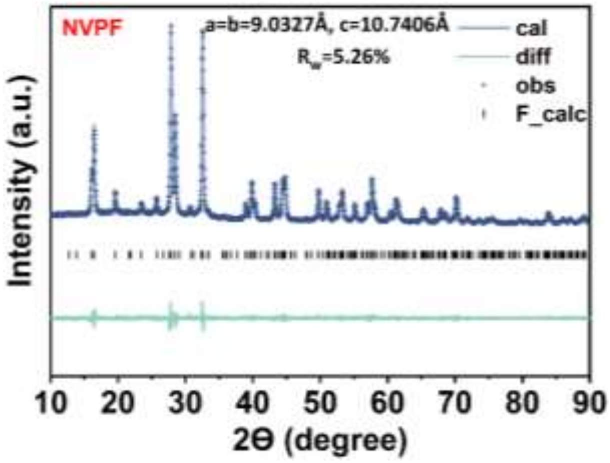

Fig. S9. The Rietveld refinement XRD spectrum of NVPF.

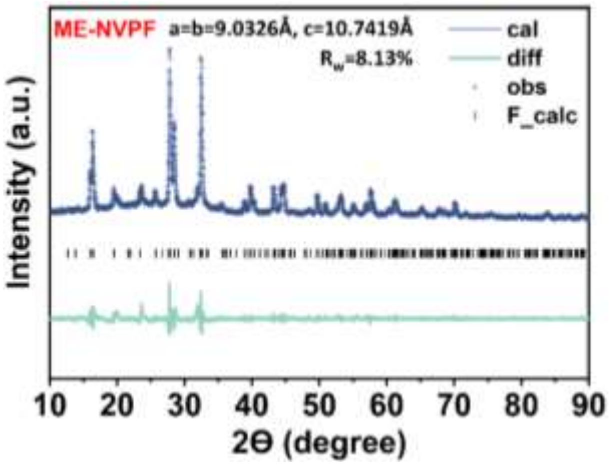

Fig. S10. The Rietveld refinement XRD spectrum of ME-NVPF.

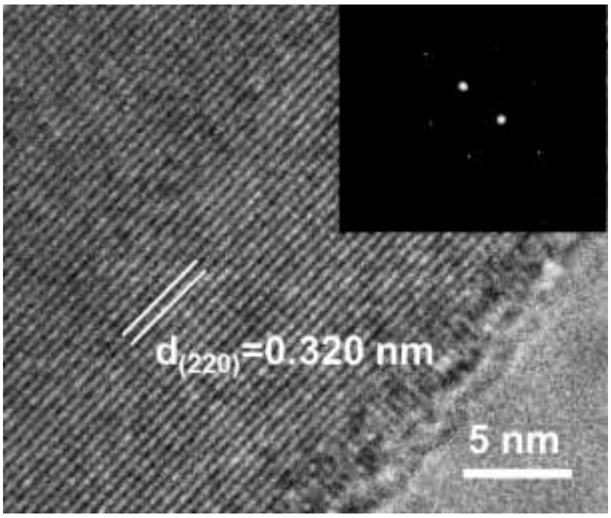

Fig. S11. The HRTEM images of HE-NVPF.

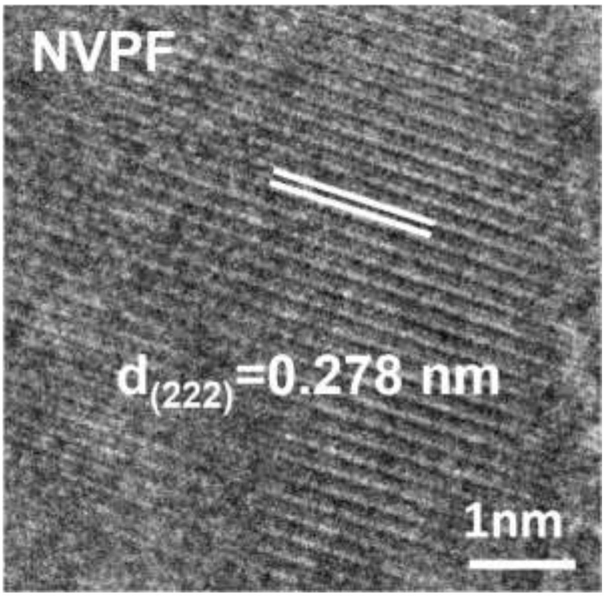

Fig. S12. The HRTEM images of ME-NVPF and NVPF.

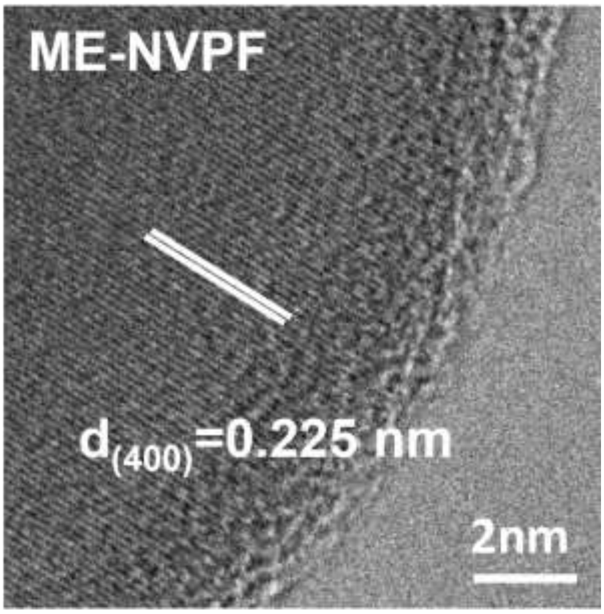

Fig. S13. The HRTEM images of ME-NVPF.

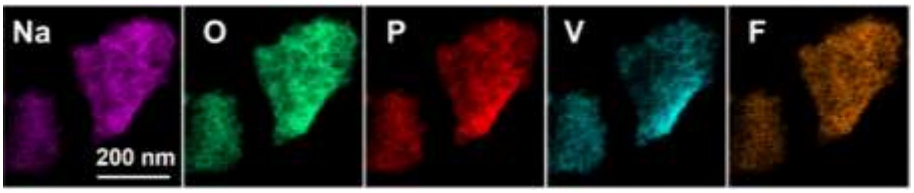

Fig. S14. The TEM-EDS images of NVPF.

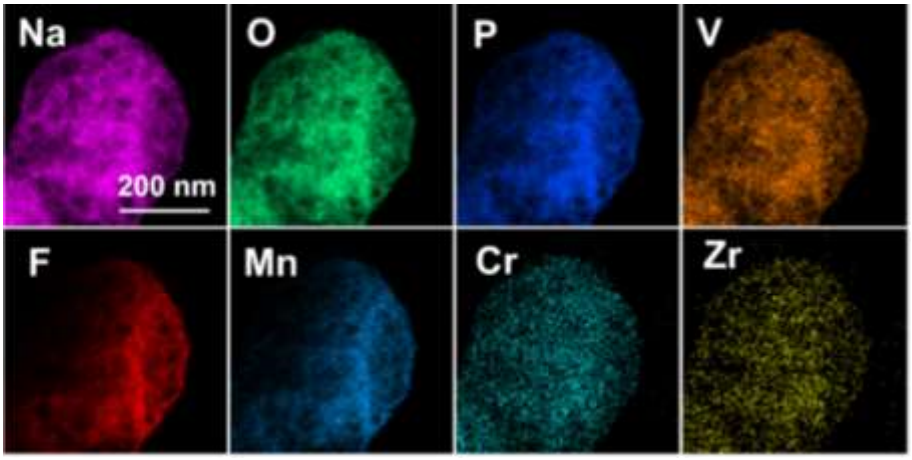

Fig. S15. The TEM-EDS images of ME-NVPF.

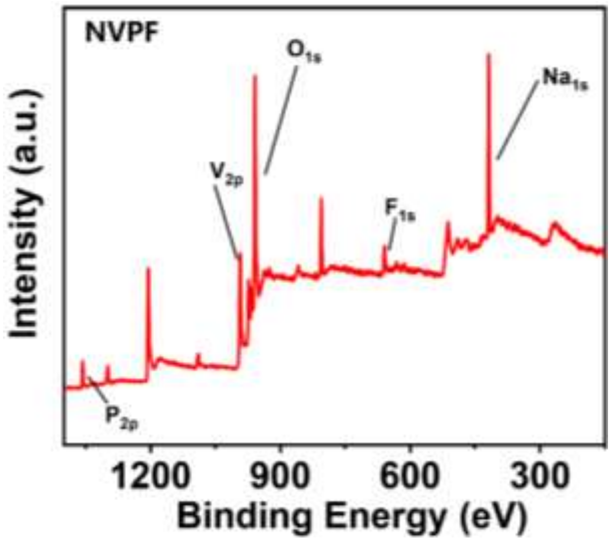

Fig. S16. The survey XPS spectrum of NVPF.

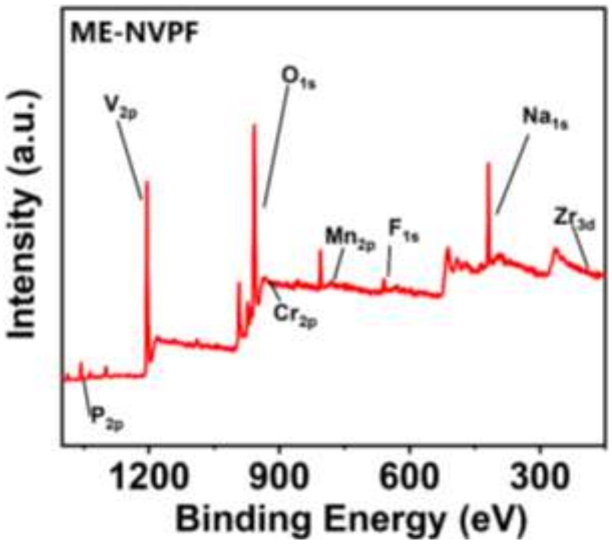

Fig. S17. The survey XPS spectrum of ME-NVPF.

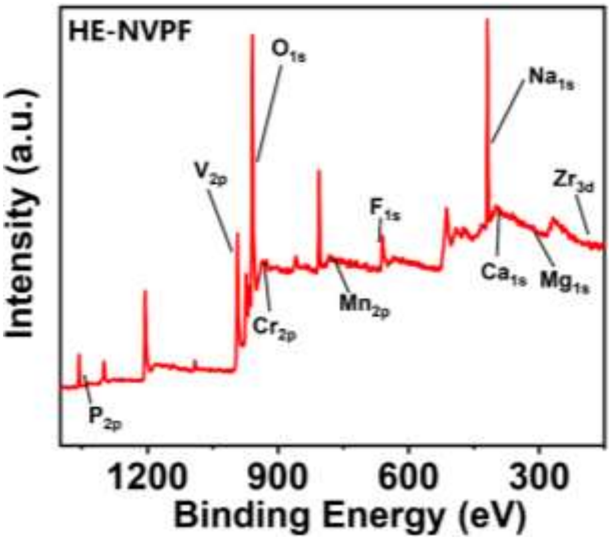

Fig. S18. The survey XPS spectrum of HE-NVPF.

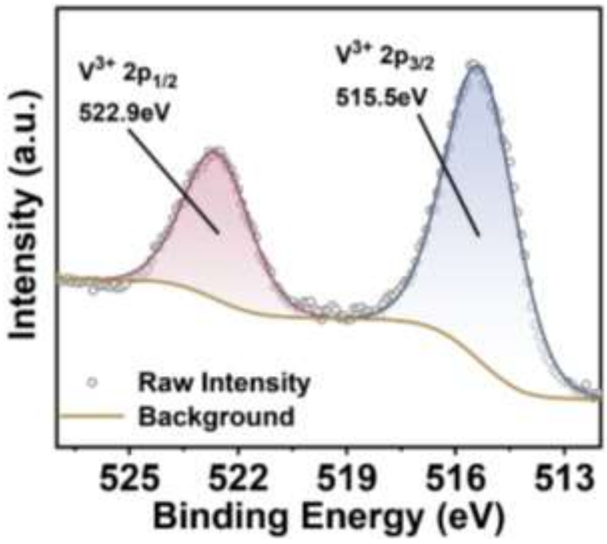

Fig. S19. The high-resolution V 2p XPS spectrum of HE-NVPF.

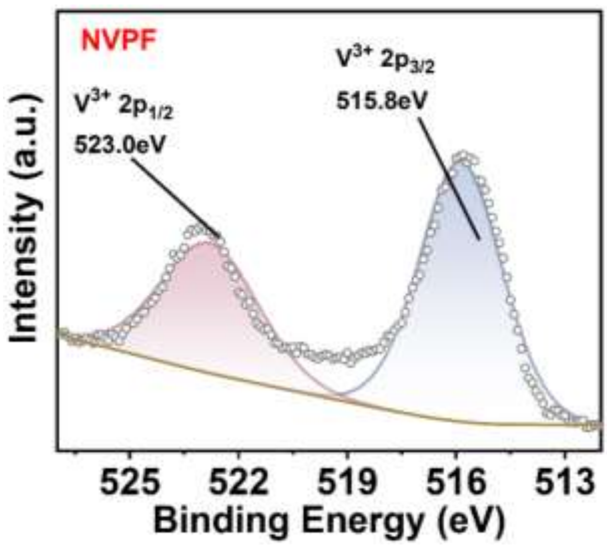

Fig. S20. The high-resolution V 2p XPS spectrum of NVPF.

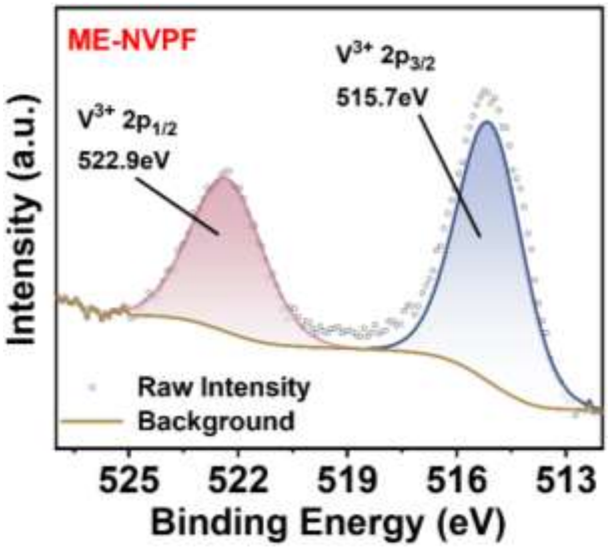

Fig. S21. The high-resolution V 2p XPS spectrum of ME-NVPF.

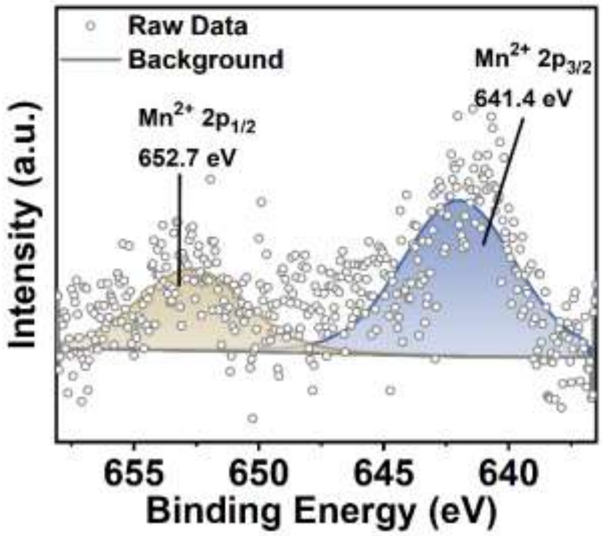

Fig. S22. The high-resolution Mn 2p XPS spectrum of HE-NVPF.

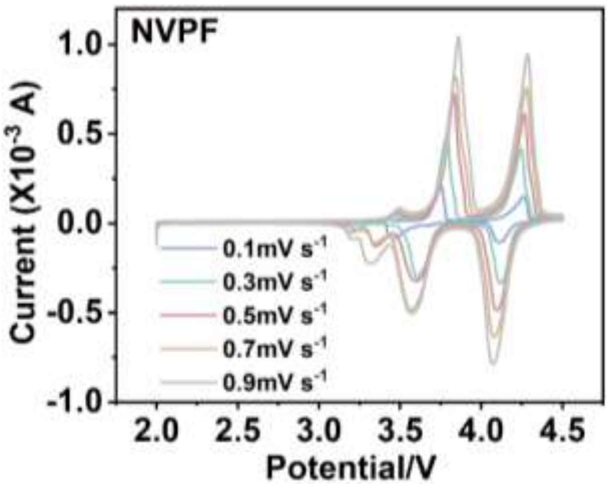

Fig. S23. The CV curves of NVPF under various scanning rates.

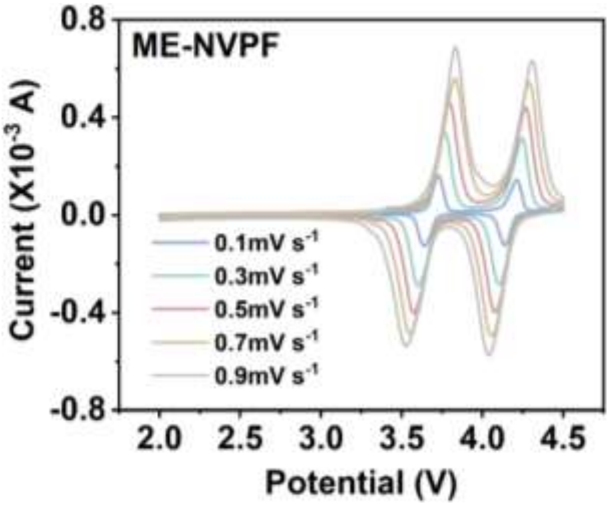

Fig. S24. The CV curves of ME-NVPF under various scanning rates.

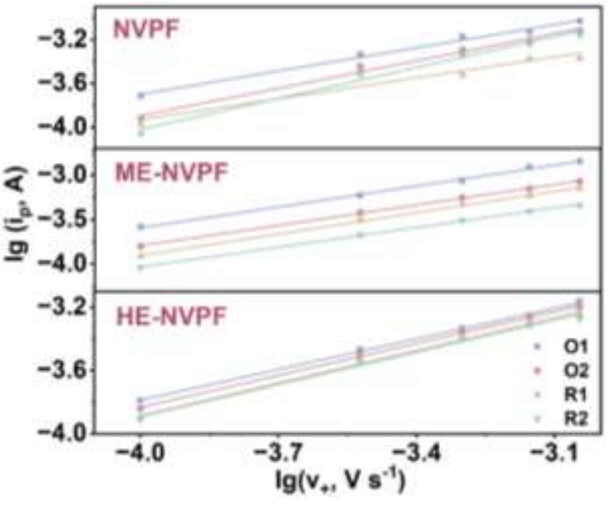

Fig. S25. The linear fittings of log (ip) and log (v) for NVPF, ME-NVPF and HE-NVPF.

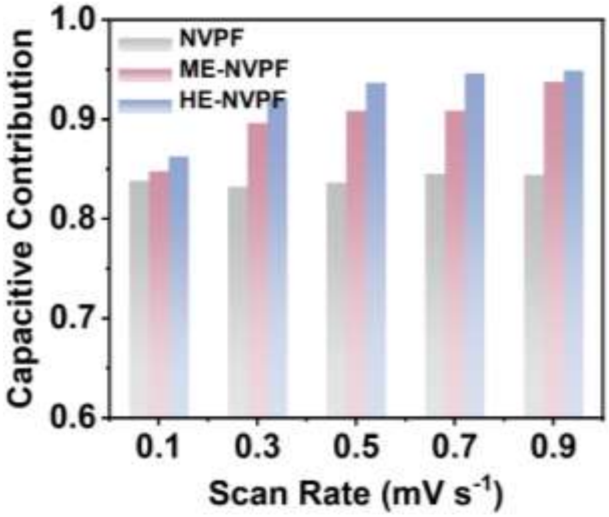

Fig. S26. The capacitive contributions of NVPF, ME-NVPF and HE-NVPF under various scanning rates.

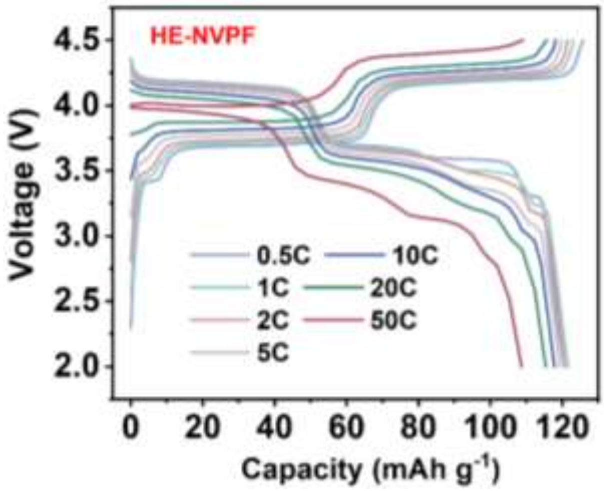

Fig. S27. The GCD curves of HE-NVPF under various current rates.

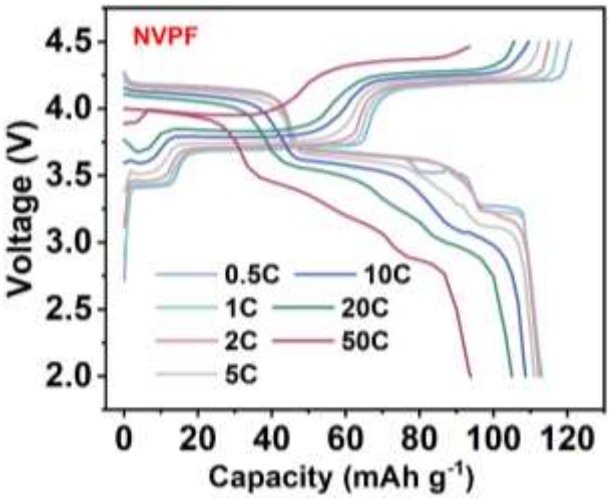

Fig. S28. The GCD curves of NVPF under various current rates.

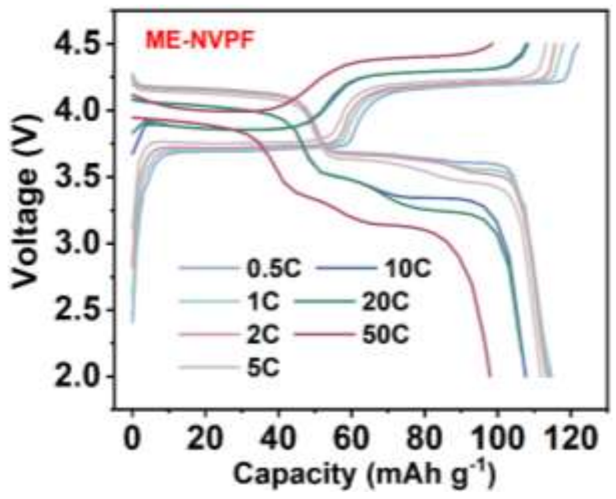

Fig. S29. The GCD curves of ME-NVPF under various current rates.

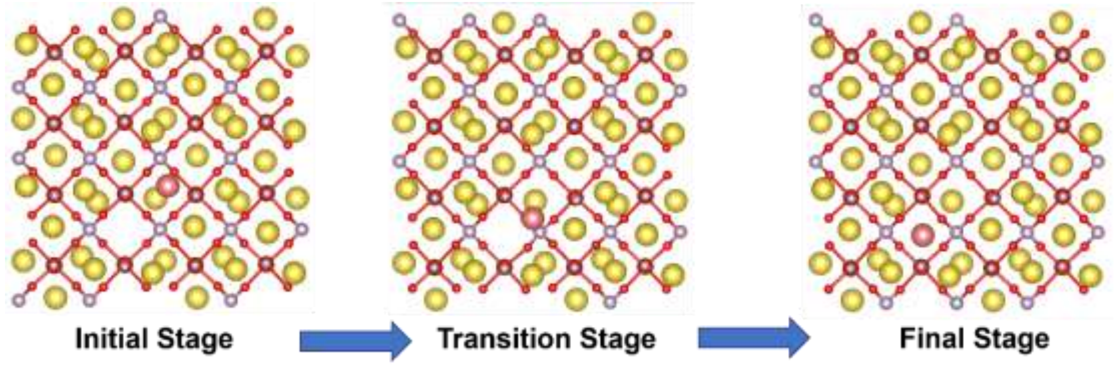

Fig. S30. The schematic illustrations of Na^+ migration pathways in NVPF under various stages.

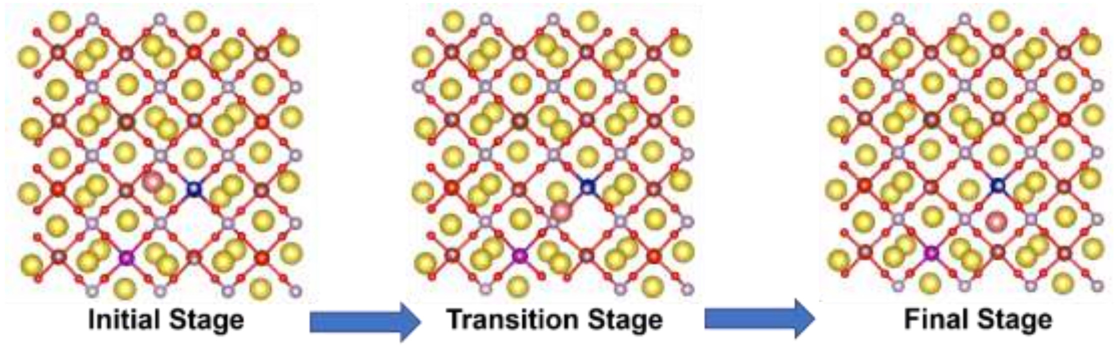

Fig. S31. The schematic illustrations of Na^+ migration pathways in ME-NVPF under various stages.

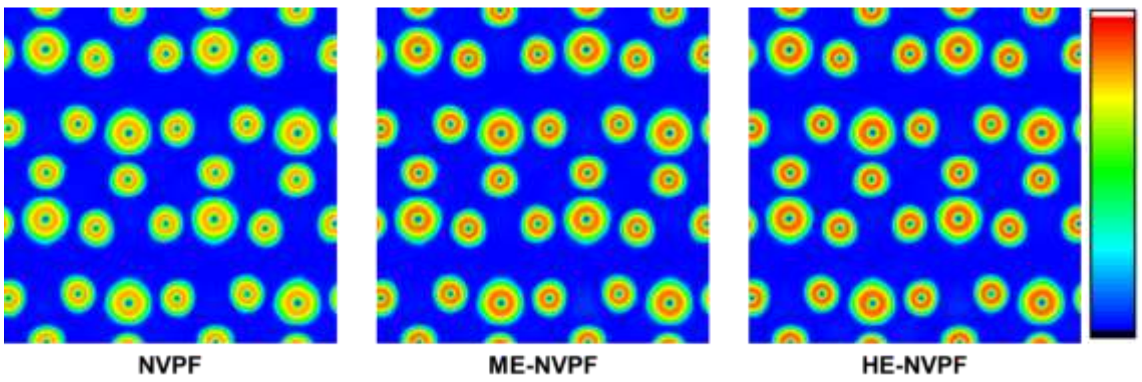

Fig. S32. The 2D contour plots of ELF of NVPF, ME-NVPF and HE-NVPF.

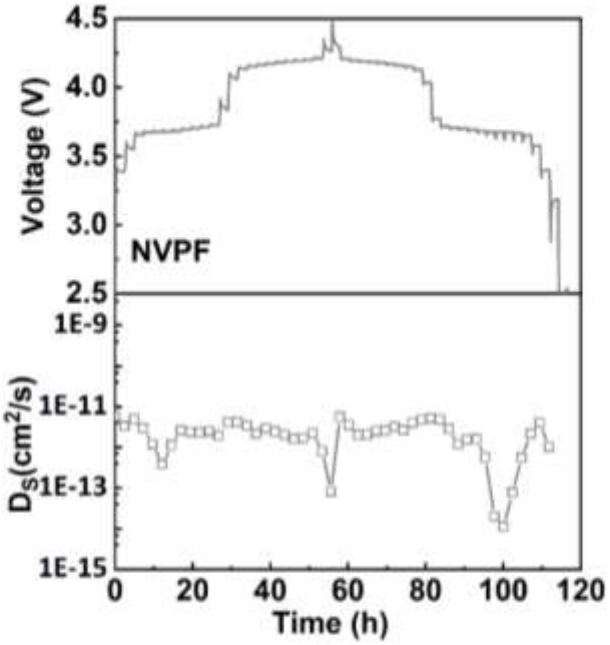

Fig. S33. The GITT curve and Na^+ diffusion coefficient of NVPF.

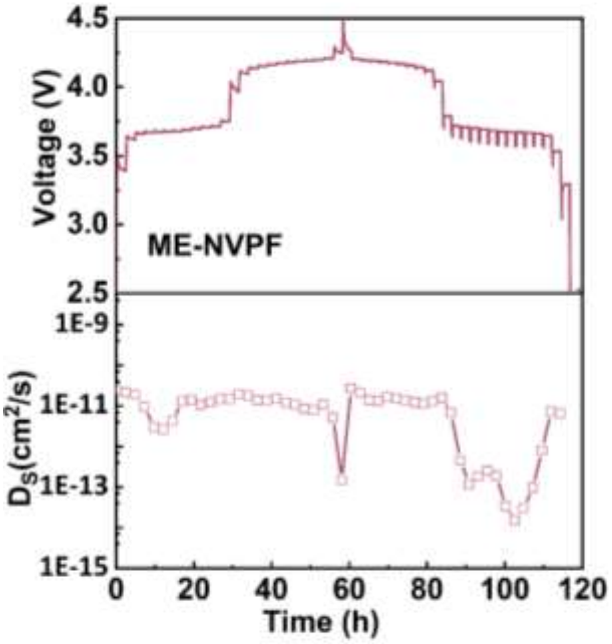

Fig. S34. The GITT curve and Na^+ diffusion coefficient of ME-NVPF.

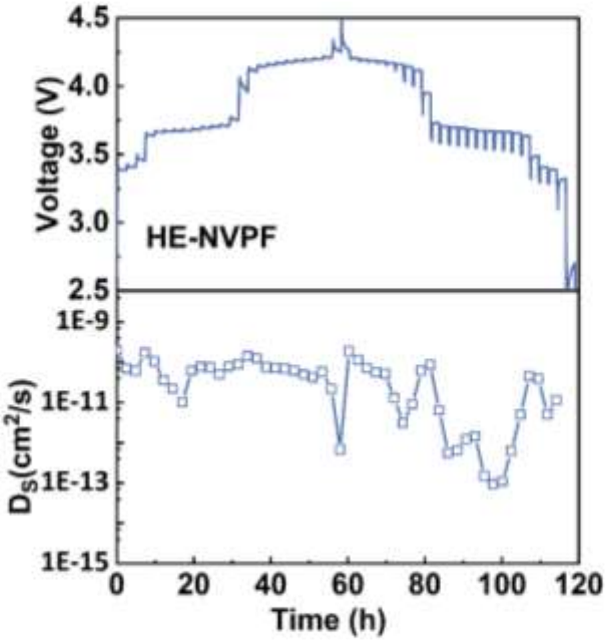

Fig. S35. The GITT curve and Na^+ diffusion coefficient of HE-NVPF.

Supplemental Equations

Equation S1.

$$\alpha=\frac{I-I_0}{I_{equ}-I_0}$$

Where α represents the extent of crystallinity, I represents the integrated intensity of the V–O–V bond peak in the Raman spectrum, I_{equ} represents the integrated intensity at saturation, and I_0 represents the initial intensity.

Equation S2.

$$\alpha = \frac{1}{1+e^{-\frac{t-a_{nucleation}}{b}}}\left(1-e^{-(k_{growth}t)^n}\right)$$

Where α represents the extent of crystallinity , $a_{nucleation}$ represents the nucleation rate constant, k_{growth} represents the growth rate constant, n represents the crystallization dimension (dimensionless), with n=3 in this model, b represents a time constant, t represents time.

Equation S3.

$$k = Ae^{-\frac{E_a}{RT}}$$

Where k represents rate constant, E_a represents the activation energy , R represents the universal gas constant, T represents temperature, A is a constant.

Equation S4.

$$2V^{(IV)}O^{2+}+14H_2O+C_2O_4^{2-} = 2[V^{(III)}(H_2O)_6]^{3+}+2CO_2\uparrow+4OH^-$$

$$2n[V^{(III)}(H_2O)_6]^{3+}+6nOH^- = [V_{2n}O_{3n}(OH)_{3n}]^{3n-}+12nH_2O$$

Equation S5.

$$i_p = kn^{1.5}AD^{0.5}Cv^{0.5}$$

Where i_p represents the peak current, n represents the electron transfer number,

A represents the effective electrode area, D represents the diffusion coefficient, C represents the electroactive species concentration, ν represents the scan rate, k is a constant.

Supplemental Tables

Table S1. Molar ratio of high-entropy elements in high-entropy $Na_3V_{1.9}M_{0.1}(PO_4)_2F_3$ libraries characterized via EDS.

Elements (Fig. 2b)	Molar ratio (%)	Elements (Fig. 2c)	Molar ratio (%)
Ca	0.093159	Mg	0.109321
Mg	0.121747	Co	0.132548
Zr	0.126015	Zr	0.111153
Cr	0.107408	Cr	0.076479
Mn	0.113704	Mn	0.108294
**Elements (Fig. 2d)**	**Molar ratio (%)**	**Elements (Fig. 2e)**	**Molar ratio (%)**
Fe	0.081457	Mg	0.110021
Ca	0.128589	Ca	0.092633
Zr	0.098278	Ni	0.148633
Cr	0.165561	Cr	0.094386
Mn	0.146349	Mn	0.078069
**Elements (Fig. 2f)**	**Molar ratio (%)**	**Elements (Fig. 2g)**	**Molar ratio (%)**
Mg	0.13138	Mg	0.11261
Cu	0.102431	Ga	0.118104
Zr	0.115566	Zr	0.117264
Cr	0.11046	Cr	0.111079
Mn	0.097636	Mn	0.09971

Table S2. Molar ratio of high-entropy elements in high-entropy $Na_3V_{1.9}(Ca, Mg, Zr, Mn, Cr)_{0.1}(PO_4)_2F_3$ characterized via ICP-OES.

Elements	Molar ratio (Measured) (%)	Molar ratio (Theoretical) (%)	Deviation (%)
**Ca**	0.1139	0.111	2.612613
**Mg**	0.122	0.111	9.90991
**Zr**	0.111	0.111	0.063063
**Cr**	0.114	0.111	2.702703
**Mn**	0.116	0.111	4.504505

Table S3. Molar ratio of high-entropy elements in high-entropy $Na_3V_{1.9}(Mg, Zr, Co, Mn, Cr)_{0.1}(PO_4)_2F_3$ characterized via ICP-OES.

Elements	Molar ratio (Measured) (%)	Molar ratio (Theoretical) (%)	Deviation (%)
**Mg**	0.124274	0.111	11.95867
**Co**	0.139444	0.111	25.62543
**Zr**	0.116506	0.111	4.959921
**Cr**	0.101437	0.111	8.615699
**Mn**	0.114114	0.111	2.804965

Table S4. Molar ratio of high-entropy elements in high-entropy $Na_3V_{1.9}(Zr, Ca, Fe, Mn, Cr)_{0.1}(PO_4)_2F_3$ characterized via ICP-OES.

Elements	Molar ratio (Measured) (%)	Molar ratio (Theoretical) (%)	Deviation (%)
**Fe**	0.11024	0.111	0.684751
**Ca**	0.101724	0.111	8.35703
**Zr**	0.115887	0.111	4.402836

**Cr**	0.113913	0.111	2.624081
**Mn**	0.126202	0.111	13.69514

Table S5. Molar ratio of high-entropy elements in high-entropy $Na_3V_{1.9}(Mg, Ca, Ni, Mn, Cr)_{0.1}(PO_4)_2F_3$ characterized via ICP-OES.

**Elements**	**Molar ratio (Measured) (%)**	**Molar ratio (Theoretical) (%)**	**Deviation (%)**
**Mg**	0.109096	0.111	1.715015
**Ca**	0.113219	0.111	1.999053
**Ni**	0.122787	0.111	10.61908
**Cr**	0.097939	0.111	11.76708
**Mn**	0.117472	0.111	5.830994

Table S6. Molar ratio of high-entropy elements in high-entropy $Na_3V_{1.9}(Zr, Cu, Mg, Mn, Cr)_{0.1}(PO_4)_2F_3$ characterized via ICP-OES.

**Elements**	**Molar ratio (Measured) (%)**	**Molar ratio (Theoretical) (%)**	**Deviation (%)**
**Mg**	0.113144	0.111	1.931812
**Cu**	0.124543	0.111	12.20086
**Zr**	0.116092	0.111	4.587325
**Cr**	0.102662	0.111	7.511396
**Mn**	0.124641	0.111	12.28957

Table S7. Molar ratio of high-entropy elements in high-entropy $Na_3V_{1.9}(Ga, Zr, Ca, Mn, Cr)_{0.1}(PO_4)_2F_3$ characterized via ICP-OES.

Elements	Molar ratio (Measured) (%)	Molar ratio (Theoretical) (%)	Deviation (%)
**Mg**	0.106881	0.111	3.710474
**Ga**	0.111838	0.111	0.7552
**Zr**	0.110253	0.111	0.673392
**Cr**	0.098382	0.111	11.36715
**Mn**	0.11342	0.111	2.179866

Table S8. Comparisons for the refined crystallographic data of NVPF, ME-NVPF and HE-NVPF obtained from XRD Rietveld refinements.

samples	a(=b)/Å	c/Å	Volume/Å^3
NVPF	9.033	10.741	876.41
ME-NVPF	9.033	10.742	876.42
HE-NVPF	9.038	10.744	877.63

REFERENCES

1. VandeVondele J, Krack M, Mohamed F *et al.* Quickstep: fast and accurate density functional calculations using a mixed gaussian and plane waves approach. *Comput Phys Commun* 2005; **167**: 103–28.

2. Hutter J, Iannuzzi M, Schiffmann F *et al.* Cp2k: atomistic simulations of condensed matter systems. *WIREs Computational Molecular Science* 2014; **4**: 15–25.

3. Gu Z-Y, Guo J-Z, Cao J-M *et al.* An advanced high-entropy fluorophosphate cathode for sodium-ion batteries with increased working voltage and energy density. *Adv Mater* 2022;**34**:2110108.

4. Perdew JP, Ruzsinszky A, Csonka GI *et al.* Restoring the density-gradient expansion for exchange in solids and surfaces. *Phys Rev Lett* 2008; **100**: 136406.

5. VandeVondele J, Hutter J. Gaussian basis sets for accurate calculations on molecular systems in gas and condensed phases. *J Chem Phys* 2007; **127**: 1-24.

6. VandeVondele J, Hutter J. An efficient orbital transformation method for electronic structure

calculations. *J Chem Phys* 2003; **118**: 4365–9.

7. Su G, Wang Y, Mu J *et al.* Insights into tiny high-entropy doping promising efficient sodium storage of $Na_3V_2(PO_4)_2O_2F$ toward sodium-ion batteries. *Adv Energy Mater* 2025; **15**: 2403282.

8. He Q, Yu B, Li Z *et al.* Density functional theory for battery materials. *Energy Environ Sci* 2019; **2**: 264–79.

9. Krukau AV, Vydrov OA, Izmaylov AF *et al.* Influence of the exchange screening parameter on the performance of screened hybrid functionals. *J Chem Phys* 2006; **125**: 224106.

10. Guidon M, Hutter J, VandeVondele J. Auxiliary density matrix methods for hartree−fock exchange calculations. *J Chem Theory Comput* 2010; **6**: 2348–64.

11. Lu T, Chen F. Quantitative analysis of molecular surface based on improved marching tetrahedra algorithm. *J Mol Graph Model* 2012; **38**: 314–23.

12. Momma K, Izumi F. VESTA: a three-dimensional visualization system for electronic and structural analysis. *J Appl Cryst* 2008; **41**: 653–8.
